# Characterization of SMA type II skeletal muscle from treated patients shows OXPHOS deficiency and denervation

**DOI:** 10.1172/jci.insight.180992

**Published:** 2024-09-12

**Authors:** Fiorella Carla Grandi, Stéphanie Astord, Sonia Pezet, Elèna Gidaja, Sabrina Mazzucchi, Maud Chapart, Stéphane Vasseur, Kamel Mamchaoui, Piera Smeriglio

**Affiliations:** 1 Sorbonne Université, INSERM, Institut de Myologie, Centre de recherche en Myologie F-75013 Paris, France.; 2 Centre de Ressources Biologiques - Myobank-AFM de l’Institut de Myologie, Hôpital de la Pitié-Salpêtrière F - 75013 Paris, France.

**Keywords:** Genetics, Muscle biology, Bioinformatics, Neuromuscular disease, Skeletal muscle

## Abstract

Spinal muscular atrophy (SMA) is a recessive developmental disorder caused by the genetic loss or mutation of the gene *SMN1* (survival of motor neuron 1). SMA is characterized by neuromuscular symptoms and muscle weakness. Several years ago, SMA treatment underwent a radical transformation, with the approval of 3 different SMN-dependent disease-modifying therapies. This includes 2 *SMN2* splicing therapies — risdiplam and nusinersen. One main challenge for type II SMA patients treated with these drugs is ongoing muscle fatigue, limited mobility, and other skeletal problems. To date, few molecular studies have been conducted on SMA patient–derived tissues after treatment, limiting our understanding of what targets remain unchanged after the spinal cord–targeted therapies are applied. Therefore, we collected paravertebral muscle from 8 type II patients undergoing spinal surgery for scoliosis and 7 controls. We used RNA-seq to characterize their transcriptional profiles and correlate these molecular changes with muscle histology. Despite the limited cohort size and heterogeneity, we observed a consistent loss of oxidative phosphorylation (OXPHOS) machinery of the mitochondria, a decrease in mitochondrial DNA copy number, and a correlation between signals of cellular stress, denervation, and increased fibrosis. This work provides new putative targets for combination therapies for type II SMA.

## Abstract 2

Characterization of SMA Type II patient muscles shows that there are changes in mitochondrial function and the amount of denervation

## Graphical abstract

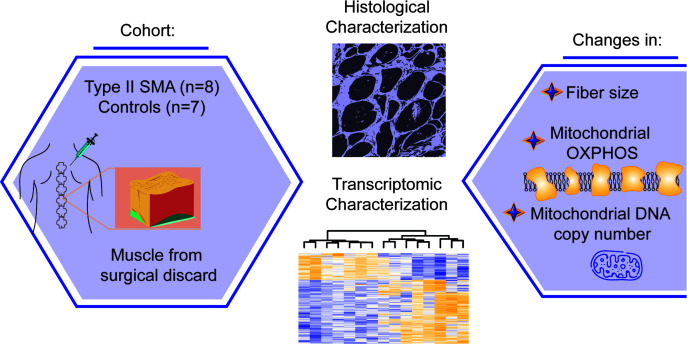

## Introduction

Spinal muscular atrophy (SMA) is a rare, autosomal, recessive, developmental disorder caused by the genetic loss or mutation of the gene *SMN1* (survival of motor neuron 1) (1). The disorder is characterized by neuromuscular symptoms, including muscular atrophy, weakness of the proximal muscles, and hypotonia (1, 2). At the cellular level, *SMN1* loss results in the death of motor neurons (MNs) that innervate the muscle. At the molecular level, SMN has a variety of associated molecular functions, including RNA splicing, R-loop resolution, and cytoskeletal dynamics (3).

SMA symptoms exist on a spectrum and patients are classified into 5 stratifications (types 0–IV) that guide clinical care (2, 4). These groups are determined based on the degree of motor symptom involvement and the age of onset. Additionally, patients are stratified by the number of copies of *SMN2*, which is the major genetic modifier of the disease. *SMN2* is a nearly identical copy of *SMN1*, situated in the same 5q13 locus that has undergone events of duplication and deletion, and is found in varying copy numbers in the human genome (5, 6). However, *SMN2* differs from *SMN1* by a few bases that modify a splice junction, inducing a low production of the full-length SMN (10%) and 90% transcription of a truncated SMN mRNA without exon 7, referred to as *SMN*Δ*7*. This transcript is unstable, has a shorter half-life, and cannot fully compensate for the function of *SMN1* (1). The importance of the dose of the SMN protein is underscored by the less severe symptoms of type II–IV SMA patients, which have increasing numbers of *SMN2* (2).

Classically, SMA has been considered a disease of motor neurons. However, as *SMN* is ubiquitously expressed throughout the body (7, 8), the tissue specificity of this effect has been difficult to understand. Emerging work has demonstrated that SMA is a whole-body developmental disorder, with SMN exerting specific roles in many tissues, including skeletal muscle (9–11). Indeed, understanding the role of SMN in non-MN tissues is necessary for clinical management. In particular, work from several groups has highlighted cell-intrinsic defects in muscle tissue in mouse models where *Smn1* is lost only in one or several muscle cell types, but not in MNs (11, 12). This work strongly suggests that restoring SMN to the muscle is critical for optimal care.

Several years ago, SMA treatment underwent a radical transformation, with the approval of 3 different *SMN-*dependent disease-modifying therapies (1), which revolutionized the life expectancy of patients. However, these treatments remain noncurative. Of these, the most efficacious is the gene therapy onasemnogene abeparvovec (ZOLGENSMA) (13), which is available as a treatment for infants with type I SMA, rendering this previous fatal disease survivable. For those patients who are not eligible for ZOLGENSMA treatment, either because of age of symptom onset, current age, or AAV immunoreactivity, 2 other treatments are available that act on the *SMN2* (1) splicing mechanism, nusinersen (Spinraza) and risdiplam (Evrysdi). Both nusinersen and risdiplam are SMN-dependent therapies whose mechanism of action is based on inducing the inclusion of exon 7 in the remaining *SMN2* copies (1, 14).

While these treatments have been life changing for SMA patients, the road ahead contains challenges for patients and clinicians because the 3 treatments do not always lead to a full restoration or alteration of clinical symptoms, partially due to the need for early treatment, putatively before symptom onset, to fully restore all SMN-related deficiency problems (15, 16). This may be partially because the levels of SMN are highest prenatally (17). However, in older children and adults with SMA, which collectively represent two-thirds of the SMA population, early gene therapy treatment is not an option, and these patients may need additional SMN-independent therapies to restore function of muscle and other tissues.

Type II SMA patients represent approximately 20% of all cases and have an onset of symptoms at between 6 and 18 months, with an unassisted life expectancy of 25 years, which has been vastly improved by the new therapies and supportive care. Most patients achieve sitting milestones, although sometimes with delay, and by clinical definition, these patients generally do not sit or walk independently. They display proximal predominant weakness, especially in the lower limbs, and most, if not all, have scoliosis (18). Due to the age of onset, clinical management includes treatment with risdiplam and nusinersen injections. However, posttreatment challenges remain, including muscle fatigue, limited mobility, and other skeletal problems, such as hip dysplasia and scoliosis (19).

Due to the rarity of tissues, there are few molecular studies on the muscle of patients with SMA, especially in those with type II SMA. In this work, we performed a histopathological and molecular characterization of paravertebral muscle from the surgical discards in a small cohort of 8 treated SMA type II patients undergoing spinal surgery for scoliosis as well as 7 non-SMA control individuals. We used RNA-seq to characterize their transcriptional profiles and correlate these with muscle histology.

## Results

### Type II SMA muscle has similar levels of SMN RNA and protein to those of scoliotic controls.

**Figure 1:**
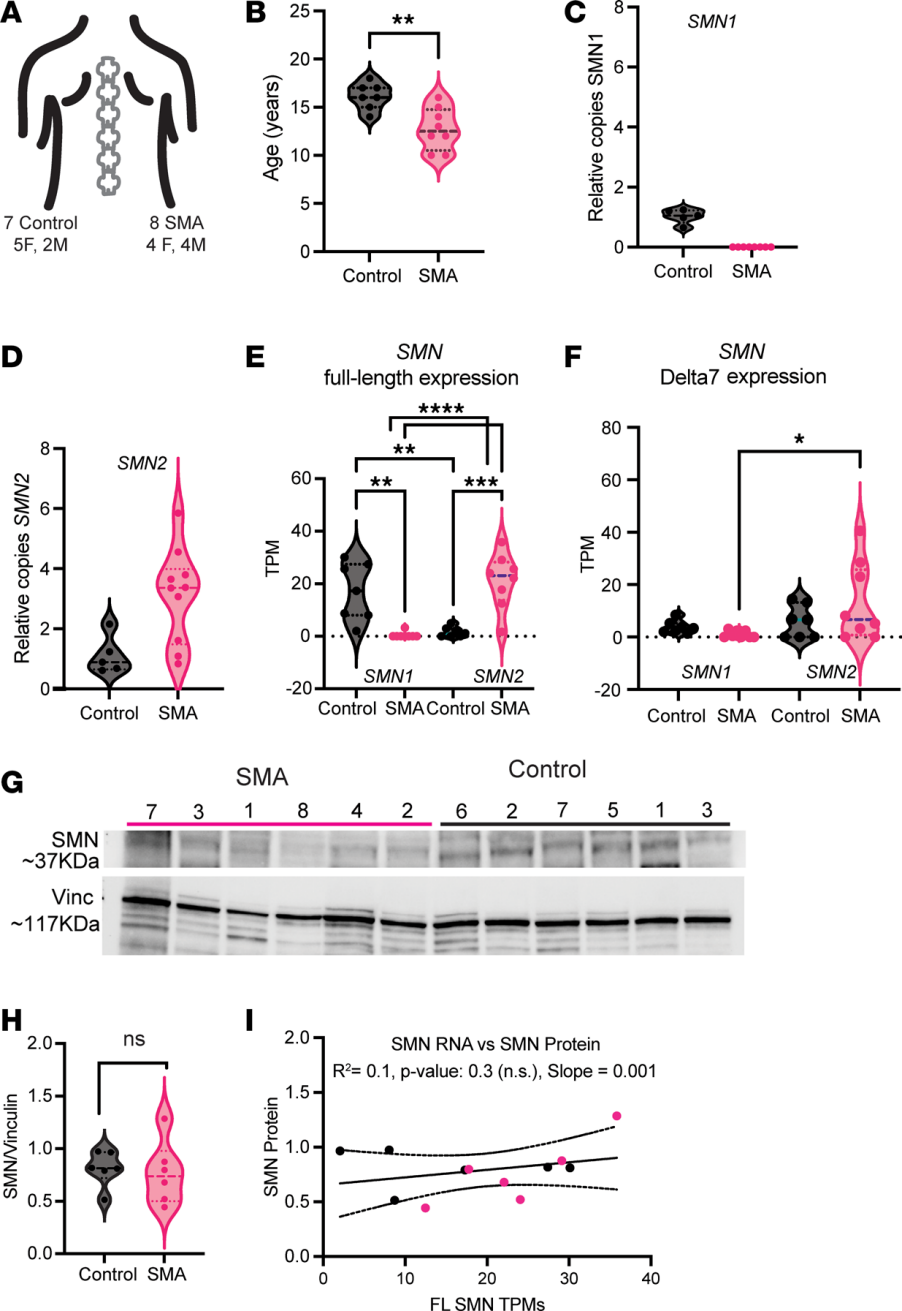
SMN RNA and protein levels are comparable between SMA type II and control paravertebral muscle. (**A**) Diagram of the cohort with the sex balance for each group. (**B**) Age, in years, for the SMA (*n* = 8) and control (*n* = 7) groups. Each data point represents 1 patient. Groups were compared with a 2-sided Student’s *t* test. **P < 0.001. (**C** and **D**) Relative quantification (qPCR) of the copy number of *SMN1* (**C**) or *SMN2* (**D**) in gDNA (*n* = 5 controls, *n* = 8 SMA). Relative quantification was calculated with *RPPH1* as a loading control. Each data point represents the average of 2 technical replicates of the measurement per patient. (**E** and **F**) Reads, represented as transcripts per million (TPM) mapping to the SMN full-length transcript (**E**), and the *SMN*Δ*7* transcript missing exon 7 (**F**). The origin of the full-length transcript, either the *SMN1* or *SMN2* locus, is designated below each pair of violin plots. Each data point represents 1 sample: *n* = 7 for controls, *n* = 8 for SMA. Adjusted *P* values are derived from an ordinary 1-way ANOVA with Dunnett’s multiple-hypothesis testing. **P_adj_ < 0.001, ***P_adj_ < 0.0001, ****P_adj_ < 0.00001. (**G**) Western blot for the approximately 37 kDa SMN protein and approximately 117 kDa vinculin protein. Each patient is labeled on the top. (**H**) Quantification of the blot in **G**. Each data point represents the normalized SMN/vinculin value for each sample (*n* = 6 controls, *n* = 6 SMA samples). The mean of the group was compared with a 2-sided Student’s *t* test. NS, not significant (*P* = 0.8). (**I**) Correlation between the TPM values of the full-length (FL) SMN transcripts in each sample in **E** and **F** compared to the normalized protein quantification in **H** (*n* = 6 controls in black, *n* = 6 SMA in pink). Pearson’s *R*^2^ value and it’s associated *P* value are reported. The solid line represents the simple linear regression and the dashed lines represent the 95% confidence internal.

To characterize the muscle of SMA type II patients, we collected a cohort of paravertebral muscle samples from patients undergoing orthopedic surgery for scoliosis. Our cohort of samples consisted of 8 type II (or type I bis) SMA patients and 7 non-SMA control individuals, also undergoing the same surgical correction for scoliosis (Figure 1A and Table 1). Muscle tissue was obtained from the surgical discard created during the surgery. To the extent possible, the cohort was sex (9 females, 6 males) and age matched, although on average, the SMA cohort is 4 years younger (mean 12.75 ± 2 years) than the control cohort (16.14 ± 1.3 years) (Figure 1B). All SMA patients received intrathecal nusinersen injections, and a subset had a clinical history of risdiplam treatment (Table 1), although the full clinical history of each sample was not always available.

As the major genetic modifier of SMA is known to be the *SMN2* gene (20), we sought to determine the relative copy number of *SMN2* in our control versus patient samples. The predicted copy number of SMN2 in type II patients is between 3 and 4 (21). However, absolute copy number detection via traditional PCR or qPCR methods can be difficult without digital PCR methods (22). Therefore, we decided to quantify the number of *SMN1* and *SMN2* copies relative to our control samples. As expected, in the control samples, we could easily measure *SMN1* copies, but detected none in the SMA samples (Figure 1C). By contrast, *SMN2* copies were present in the SMA type II samples in variable relative amounts, with 3 out of 8 patients having about the same levels as control samples (Figure 1D), while most samples (5 out of 8) had more, suggesting a gene conversion event with *SMN1*. However, it is important to note that *SMN2* copy number naturally varies in the population.

We performed RNA-seq of the same muscle samples to characterize their transcriptome. We began by analyzing the reads mapping to either the full-length or *SMN*Δ*7* (6) transcripts. Using a variety of SNPs in the introns and UTR regions of the 2 SMN copies (23, 24), we were able to annotate transcripts derived from either the *SMN1* or *SMN2* copy. As expected, when comparing the full-length canonical SMN transcripts, we observed a dramatic decrease between control and SMA samples, where few to no reads were mapping to *SMN1*, consistent with the SMA diagnosis and our gDNA qPCR (Figure 1E). In control samples, we observed few full-length SMN reads mapping to *SMN2*, with the plurality of *SMN2* mapping reads resulting in the *SMN*Δ*7* transcript (Figure 1, E and F). However, in the SMA patients, we observed many more full-length than *SMN*Δ*7* transcripts deriving from the *SMN2* locus (Figure 1, E and F). We hypothesize that this may be due to treatment with risdiplam, but this cannot be concluded without pre- and posttreatment samples. To validate the SMN mapping approach, we used the same pipeline to map reads from a previous RNA-seq study that profiled the biceps muscle of Duchenne muscular dystrophy (DMD) and SMA type I patients (25, 26). We observed few to no reads coming from the *SMN1* locus in SMA type I patients and an increase in *SMN*Δ*7* transcript from the *SMN2* locus (Supplemental Figure 1A; supplemental material available online with this article; https://doi.org/10.1172/jci.insight.180992DS1). However, in these untreated samples, there was no significant increase in full-length splicing from the *SMN2* allele (Supplemental Figure 1A), further leading us to hypothesize this may be an effect of the risdiplam treatment in our patients. Additionally, a higher *SMN2* copy number may be responsible for the increased number of full-length *SMN2* transcripts, but we observed no correlation between these 2 parameters (Supplemental Figure 1B). This is in line with previous findings in the spinal cord that *SMN1* and *SMN2* copy number correlate poorly with protein expression (17).

No correlation was observed between the amount of *SMN2* canonical transcript and the amount of *SMN*Δ*7* transcripts (Supplemental Figure 1C) — rather, 3 out of 8 SMA samples had high *SMN*Δ*7* levels, irrespective of the amount of *SMN2* full-length transcript. We also tested the level of RNA expression with age, as SMN levels have been shown to decrease in aging (17); however, within our limited age range, no decrease was observed (Supplemental Figure 1D). Previous studies have suggested that the amount of SMN full-length transcript is also dependent on the levels of 2 splicing factors, hnRNPA1 and hnRNPA2 (27, 28). Our RNA-seq data confirm that global expression levels of *HNRNPA1* and *HNRNPA2B1* correlate with increased levels of total *SMN*Δ*7* observed (Supplemental Figure 1E). Consistent with the role of SMN in splicing, we observed a higher degree of *SMN* transcripts with retained introns in the SMA samples compared with controls (Supplemental Figure 1F).

We next determined the level of the full-length, approximately 37 kDa SMN protein in the control and SMA samples using Western blotting. On average, SMA patients had similar levels of SMN protein to the scoliotic controls (Figure 1, G and H). We next correlated the RNA and protein levels and observed no correlation between the two (Figure 1I). Collectively these data allow us to conclude that in this small cohort, despite some variation in copy number and RNA levels, at adolescence the full-length SMN protein levels were similar in the SMA and control groups. This lack of observable difference may be due to the low levels of SMN protein that are observed after birth (17).

### SMA muscles have altered myofiber size, with the presence of multiple internalized nuclei in a single fiber.

**Figure 2:**
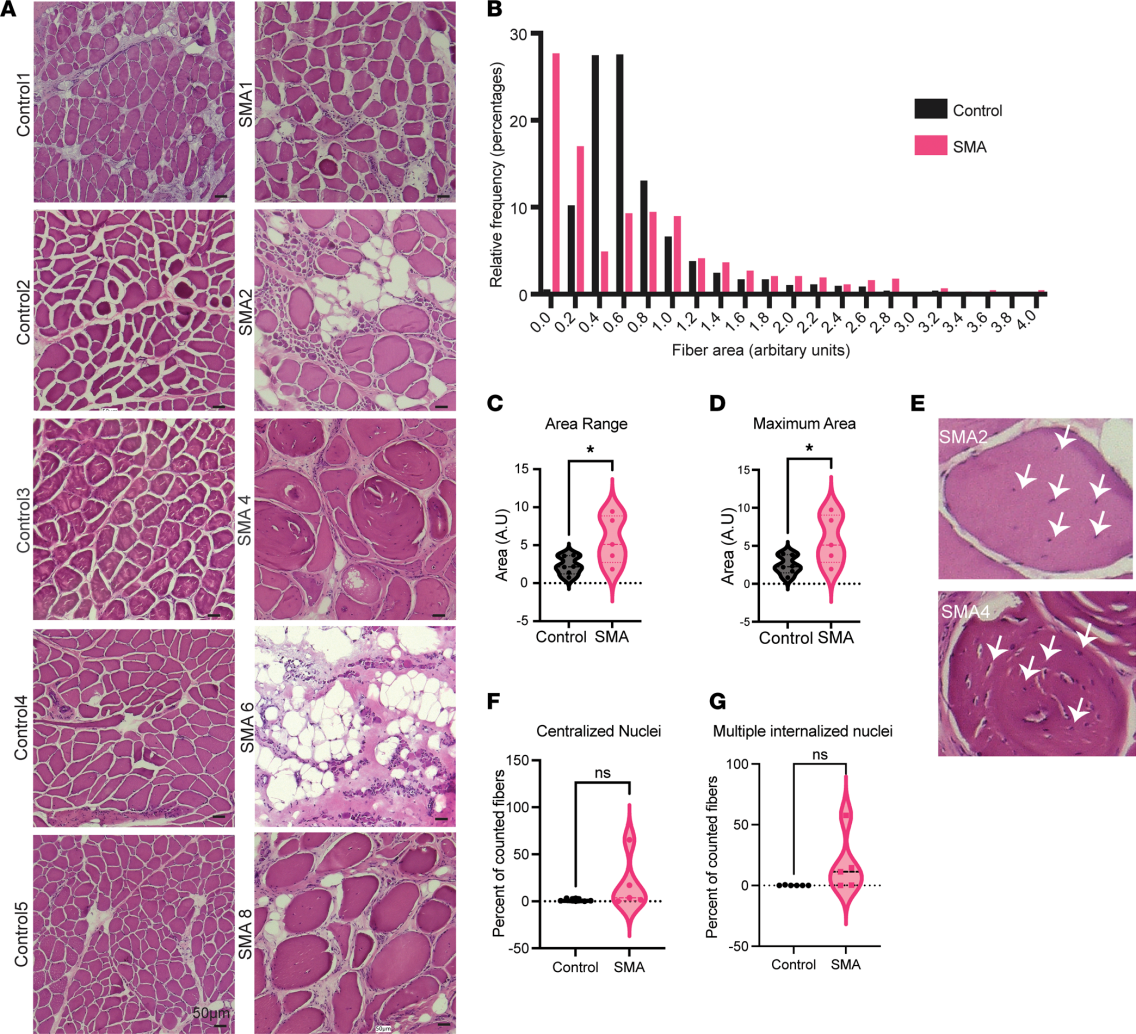
SMA type II paravertebral muscle after treatment is characterized by abnormal myofiber size distribution. (**A**) H&E staining of muscle tissues from control and SMA patient samples. Representative images (3–5 images were acquired per sample) are shown for each group. Original magnification, ×200. Scale bars: 50 μm. (**B**) Histogram of the fiber area. The frequency distribution represents the percentage of all fibers per group that falls within the bin area. Fiber area was measured in arbitrary units. *n* = 6 controls and *n* = 5 SMA, with multiple fiber measurements per sample, taken from 1 representative image per sample. (**C**) The range (highest to lowest) of fiber area measurements per patient. Each data point represents 1 image of 1 patient sample (*n* = 6 controls, *n* = 5 SMA). Group averages were compared using a 2-sided Student’s *t* test. *P < 0.05. (**D**) The maximum fiber area measured for each patient from 1 representative image. Each data point represents 1 patient (*n* = 6 controls and *n* = 5 SMA). Group averages were compared using a 2-sided Student’s *t* test. *P < 0.05. (**E**) Representative cropped and enlarged myofibers from images showing myofibers with multiple internalized nuclei from the 2 patients where we observed this phenomenon. Internalized nuclei are designated by white arrows. (**F** and **G**) Quantification of single centralized (**F**) or multiple internalized nuclei (**G**) in SMA (*n* = 5) and controls (*n* = 6). Each data point represents the percentage of such nuclei in the fibers counted in 1 image from each patient sample. Group averages were compared using a 2-sided Student’s *t* test. NS, not significant (*P* > 0.05).

We next turned our attention to characterizing the muscle histologically. We performed H&E staining for the samples with well-defined muscle fiber architecture (5 out of 8 SMA patients). H&E staining demonstrated several classic signs of SMA muscle histology, including the presence of hypertrophic fibers in 2 samples, SMA4 and SMA8 (Figure 2A), and the clusters of small fibers suggestive of denervation in samples SMA2 and SMA6 (Supplemental Figure 2A). The concurrent presence of both atrophic and hypertrophic fibers has been previously noted in SMA type III muscle (29). We quantified the area of each myofiber and plotted the distribution of sizes for the control samples and SMA samples (Figure 2B). While the control samples had myofibers with relatively homogeneous areas, the range of the area measurement (Figure 2C) as well as the maximum area was increased in the SMA samples (Figure 2D). Moreover, in addition to the within-sample variation in muscle fiber size in the SMA patients, we also observed a patient-to-patient variability in fiber size that was less prominent among controls (Supplemental Figure 2B).

Next, we assessed the percentage of the myofibers with internalized nuclei, a morphological feature common to several myopathies (30). Centralization of nuclei occurs as a response to injury during satellite cell fusion in fiber regeneration. Thus, observing centralized/internalized nuclei in a myopathy can imply that the tissue is attempting to repair itself. We first scored the slides for fibers with internalized nuclei and observed an increase in some, but not all, SMA samples. In some samples, particularly in SMA2, -4, and -8, we observed several myofibers with multiple internalized nuclei (Figure 2, E and F). However, this was not present in all samples. Within a single tissue section, we could observe both fibers with and without these multiple internalized nuclei in the same cluster, and in general, these multicentralized nuclei were found in the largest fibers.

Despite the limitations of the sampling method, namely, that we are viewing a small portion of a large muscle, this histological analysis showed that in SMA samples, there is a disorganization of the muscle fiber architecture, with a high range of variability in fiber size both within a single patient and between patients.

### RNA-seq of SMA paravertebral muscle reveals changes in calcium regulation and oxidative phosphorylation.

**Figure 3:**
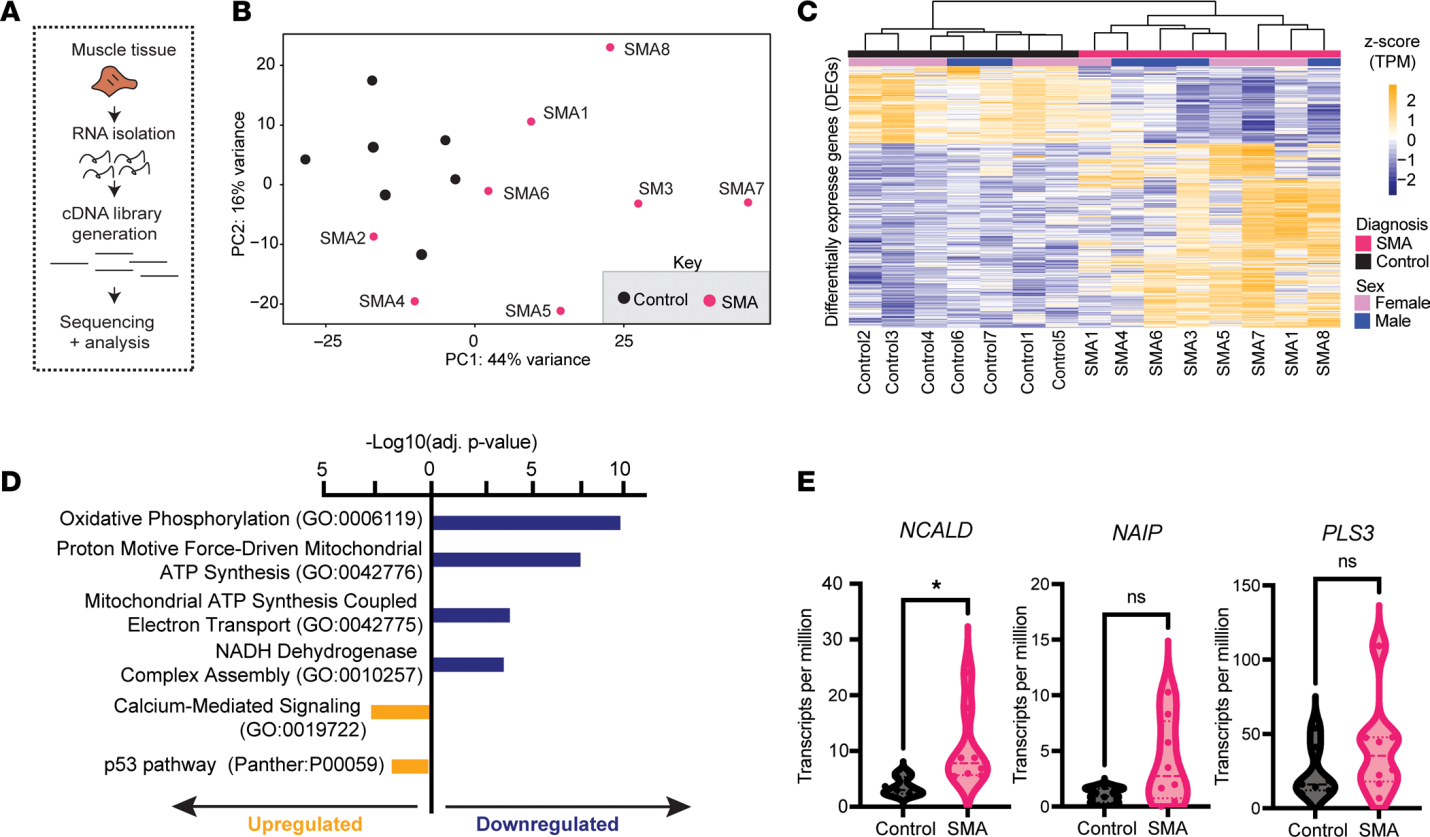
Transcriptional characterization of treated SMA and control muscle samples. (**A**) Diagram of the RNA-seq library generation process used for the samples. (**B**) Principal component analysis (PCA) plot obtained using the top 2,000 variably expressed genes in each muscle RNA-seq library. Each data point represents the cDNA library of a single sample. The variance explained for each principal component (PC) is plotted on the axes. (**C**) Heatmap of the genes that are differentially expressed (DEGs) between SMA and control samples. Each column represents the DEGs from 1 sample, which have been hierarchically clustered. Diagnosis and sex are designated by the colored bars on the top. Transcripts on the heatmap are presented as *z*-scored transcripts per million (TPM) counts. DEGs include 166 downregulated and 396 upregulated genes comparing SMA- versus control-derived samples using DESeq2 using the parameters log_2_(fold change) > 0.5, *P*_adj_ < 0.05, and standard error estimate for the log(fold change) standard error (lfcSE) of 1. Genes, fold change, and the *P*_adj_ values for all DEGs can be found in Supplemental Table 1. (**D**) GO terms associated with the upregulated and downregulated genes. The terms are plotted according to their *P*_adj_ value of enrichment. (**E**) TPM of 3 well-known SMA modifier genes, *NCALD*, *NAIP*, and *PLS3*. Each data point represents a single patient. The means of each group were compared with a 2-sided Student’s *t* test. *P < 0.05. NS, not significant (*P* > 0.05).

To obtain a broad molecular picture of the state of the SMA muscle, we performed RNA-seq of each sample (Figure 3A). Principal component analysis highlighted the heterogeneity in the samples, reflecting what we observed in our histological characterization, with approximately 44% of the variation being explained by PC1 that captured the diagnosis (i.e., SMA vs. control) (Figure 3B). Differential expression analysis found 166 downregulated genes and 396 upregulated genes (Figure 3C and Supplemental Table 1). Pathway analysis showed an enrichment for mitochondrial processes such as oxidative phosphorylation (OXPHOS) and the citric acid cycle (TCA) among the downregulated genes, and an enrichment of calcium signaling and P53 target pathways among the upregulated genes (Figure 3D). We next sought to determine whether we could observe any sex-specific effects in our samples, as within the SMA cohort we had an equal number of both sexes (XX, XY, *n* = 4). We also performed this comparison with the control samples. However, only the classic X- or Y-linked transcripts were differentially expressed, including *XIST* and *TSIX* upregulated in the female SMA muscle and *TXLNGY* and *DDX3Y* upregulated in the male samples (Supplemental Table 2).

As is the case for complex diseases, rare monogenic diseases can also be modulated by further genetic factors, giving rise to variability in the phenotypes presented (31). Several genetic modifiers have been described for SMA, and a handful of these have been validated in human samples (32–34). We observed an increase in *NCALD* expression in the SMA samples compared with controls, despite a broad range of its expression (Figure 3E). Previous studies have reported that a decrease in *NCALD* expression was associated with milder SMA phenotype (35), suggesting that the increased expression of *NCALD* is a detrimental compensatory mechanism to the loss of SMN. By contrast, we did not observe an increase in *NAIP* or *PLS3* levels (Figure 3E) (36).

MicroRNAs (miRNAs) have also been shown to have an important role in shaping the transcriptome. Many miRNAs have well-described roles in muscle development, and are aptly named myoMiRs (37–39). Perhaps the best established of these is the role of miR-206 in skeletal muscle maturation (40, 41). Furthermore, many studies in SMA have highlighted the essential role of changes of miRNAs to the SMA phenotype, and these miRNAs have also been proposed as biomarkers of nusinersen response and disease progression (38, 42–47). We used miRNA amplification and probe sets to measure the levels of 3 important myoMiRs that have been implicated in SMA and in muscle gene regulation: miR-24, miR-1, and miR-206. No difference was observed in the levels of miR-24 and miR-206. However, the levels of miR-1 were significantly decreased in the SMA samples (Supplemental Figure 2C). This is in line with a recent study that found that SMN, in combination with MYOD, can regulate the expression of miR-1 (42). However, we observed this decrease despite detecting similar levels of SMN protein (Figure 1H), suggesting additional levels of regulation. Of miR-1’s 1,349 target genes predicted by miRDB (see Methods), 52 were among our upregulated genes, including *SLC24A2*, *SYT1*, and *SHANK2*, which are involved in synaptic function (Supplemental Figure 2D and Supplemental Table 3).

Collectively, we established the landscape of altered transcripts in type II SMA muscles. Furthermore, the transcriptome of the SMA patients was more heterogeneous than that of the control individuals despite deriving from similar surgical conditions.

### SMA type II muscle shows histological and transcriptional hallmarks of denervation.

**Figure 4:**
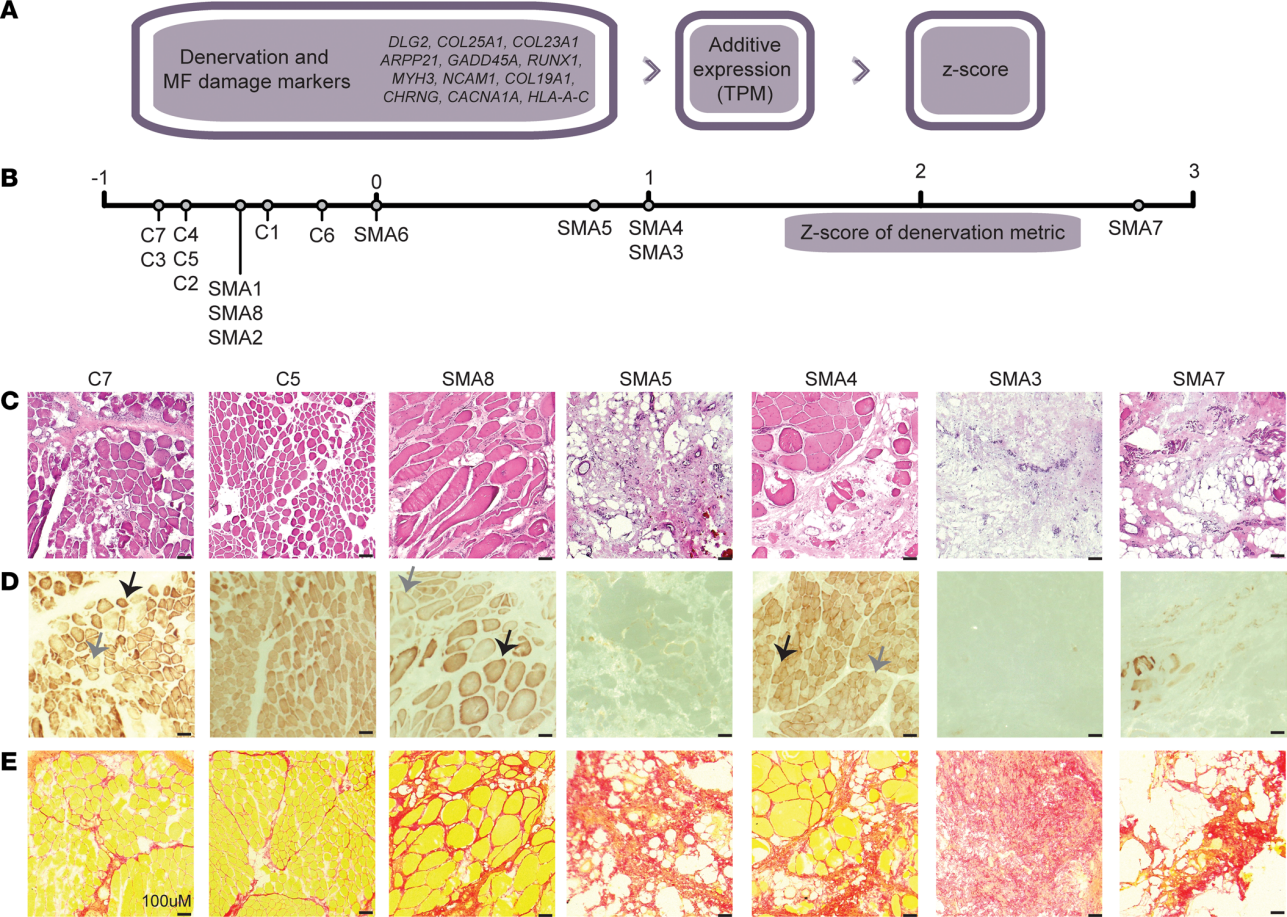
SMA type II muscle shows histological and transcriptional hallmarks of denervation. (**A**) Schematic of how the denervation score was calculated for each sample, using the expression (in TPM) of the highlighted genes. MF, myofiber. (**B**) The denervation score for each sample represented on a number line. (**C**) H&E images of selected control and SMA samples. (**D**) Cytochrome oxidase activity staining. Fibers with dark brown staining represent areas with high enzymatic activity, generally associated with type I fibers. Example fibers are highlighted by black arrows for those with dark staining (type I) and light staining (type II). (**E**) Sirius red–stained images of control and SMA muscle samples. Red staining occurs predominantly on areas with large amounts of collagen I, i.e., fibrotic areas. Scale bars: 100 μm (**C**–**E**).

We hypothesized that the transcriptional heterogeneity could derive from the onset of symptoms and the timeline of treatment, as each patient might have a different degree of denervation. However, many of the clinical metadata about these samples was unavailable. Therefore, to better understand the dynamics at play, we referenced a recent atlas of mouse muscle denervation (48), which had scored the genes most characteristic of denervation in type I fibers. Using a set of 14 genes (Figure 4A), we developed a *z*-scored denervation grade based on the expression of these markers (see Methods) and scored each of the control and SMA samples. The homogeneous scoring of the control samples compared to the variability in the SMA samples mirrored what we saw in the whole transcriptome (Figure 4B). We could also observe this same trend in the histology, where SMA patients with a score closer to zero, such as SMA8, showed a better formed fiber structure by H&E, similar to what was seen in the controls (Figure 4C).

Denervation can also affect fiber type in the muscle or cause a shift between fiber types. Previous studies on SMA patients have found a loss of type II glycolytic fibers (49), which mirrors what is seen in mouse models of SMA (11, 50, 51). However, in all these studies, muscle with a higher starting fraction of type II fibers were investigated. By contrast, studies have shown that in paravertebral muscle, 74% of the fibers are type I (slow) in the superficial and deep thoracic regions and that in the lumbar region, and 57% of the fibers are type I in the superficial muscles versus 63% type I in the deep muscles (52).

To assess changes in fiber type in our cohort, we employed 2 methods. Due to the quality of the muscle sections, we were not able to perform classic antibody-based IHC or immunofluorescence methods to detect fiber types. Therefore, we utilized a bulk RNA-seq deconvolution method, where bulk RNA-seq data can be used to predict the fiber type in the original tissue sample, based on the expression profiles of type I (slow) and type II (fast) muscle fibers derived from single-cell sequencing profiles (53). In accordance with the previous findings, fiber type deconvolution showed that in control samples, we had an average of 66% type I fibers (Supplemental Figure 2E). Similarly, SMA samples had an average of 69% type I, suggesting that RNA expression of overall fiber type markers was not highly affected. Indeed, plotting the changes in myosin expression showed a global decrease in abundance compared with controls, but not in type (Supplemental Figure 2F). Next, we used cytochrome oxidase staining (54). Cytochrome *c* oxidase is an enzyme that is found in the electron transport chain (ETC) in mitochondria. Intense dark brown is associated with type I fibers due to their large number of mitochondria. In control samples, most fibers stained dark brown, indicative of the expected predominance of type I fibers (Figure 4D). In the SMA samples, however, we observed a wide heterogeneity of staining patterns, with some that were highly similar to controls (patients 4 and 8), and other slides with no staining (SMA3, -5, and -7) (Figure 4D). Overall, this generally followed the trend of the denervation scoring, although an important caveat is that storage conditions can affect enzymatic activity.

As denervation is associated with muscle fibrosis (55), we sought to assess the degree of muscle fibrosis using Sirus red staining. In 3 of the samples with little to no fiber structure, we noticed especially prominent Sirius red staining (Figure 4E; SMA3, -5, and -7), while others had staining very similar to controls (Figure 4E, SMA8). We quantified and scored the fibrosis staining (Table 2) and found a correlation between the denervation and fibrosis scores (Supplemental Figure 2G), but not with the amount of full-length *SMN* transcript (Supplemental Figure 2H).

In summary, we validated a transcriptional signature that correlated with classic histological signs of denervation, including changes in fiber size and increase in fibrosis, and saw that these characteristics were variable among our 8 samples, although the clinical reasons for this remain unclear.

### Mitochondrial ETC complex expression and mtDNA copy number are altered in type II SMA muscle.

**Figure 5:**
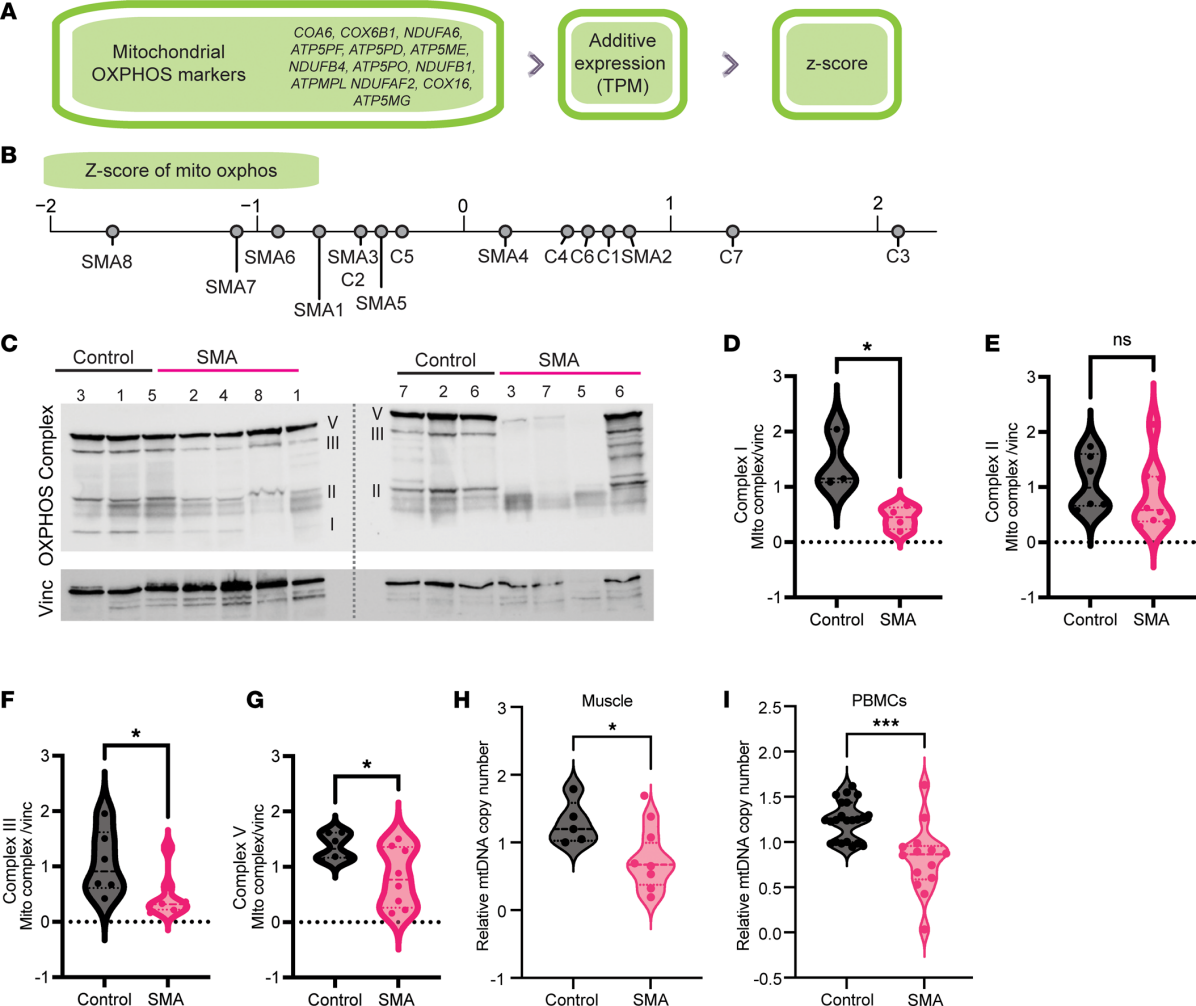
Mitochondrial electron transport chain (ETC) complex expression and mtDNA copy number are altered in type II SMA muscle. (**A**) Schematic of how the mito-score was calculated for each sample, using the expression (in TPM) of the highlighted oxidative phosphorylation (OXPHOS) genes. (**B**) The mito-score for each sample represented on a number line. (**C**) Western blots of control (*n* = 6) and SMA (*n* = 6) muscle samples incubated with the anti-OXPHOS antibody cocktail and vinculin as the housekeeping gene. The dotted line represents the 2 individual Western blots, imaged at the same time. Each band represents an ETC complex, which is labeled. Complex IV was not detected in either blot. Complex I was not detected in the blot on the right. The proteins designating each complex are as follows: complex I, NDUFB8; complex II, SDHB; complex III, UQCRC2; complex IV, MTCO1; and complex V, ATP5A. (**D**–**G**) Quantification of the blots in **C**. Each data point represents the normalized complex/vinculin value for each sample (*n* = 6 controls, *n* = 8 SMA samples). (**D**) Complex I, where *n* = 3 control and *n* = 4 SMA samples. (**E**) Complex II. (**F**) Complex III. (**G**) Complex V. The means of each group were compared with a 2-sided Student’s *t* test. *P < 0.05. NS, not significant (*P* > 0.05). (**H**) Relative quantification (qPCR) of the mtDNA copy number normalized to the numbers of gDNA copies using the β2-microglobulin gene in control (*n* = 5) and SMA (*n* = 8) paravertebral muscle gDNA samples. Each data point represents 1 sample. The means of the groups were compared with a 2-sided Student’s *t* test. *P < 0.01. (**I**) Same as in **H**, but from gDNA derived from the PBMCs of type III SMA patients. Each data point represents 1 sample (*n* = 22 controls, *n* = 14 SMA). Groups were compared with a 2-sided Student’s *t* test. ***P < 0.001.

The major pathways we observed downregulated in the SMA muscles samples were related to mitochondrial metabolic capacity, and particularly OXPHOS (Figure 3D). Using the MitoCarta gene list (56), we compared our differentially expressed genes and found 42 genes overlapping (Supplemental Figure 3A), with many of these related to proteins of the ETC that are responsible for OXPHOS (Supplemental Figure 3, B and C, and Supplemental Table 4). As we had previously observed that our small cohort is heterogeneous, we decide to calculate a mitochondrial OXPHOS score using a panel of 13 genes for each sample to better capture sample-to-sample variation (Figure 5A). As more expression of OXPHOS markers is associated with healthier muscle, given the large number of mitochondria present in type I fibers, SMA samples generally had negative *z* scores (Figure 5B). Although we hypothesized a negative correlation between the denervation score and the mito-score, samples with lowest mito-scores, for example SMA8 (score –1.7), did not always have the highest denervation scores (score–0.4) or the highest fibrosis scores (score 0.02) (Supplemental Figure 3, D and E, and Table 2). These discrepancies can highlight complexities in the biology, or difficulties in the sampling.

To better assess the loss of the OXPHOS-complex members, we performed Western blotting on our patient samples with an antibody cocktail that can recognize 1 member of each complex (Figure 5C). Likely due to its hydrophobic nature, we were consistently unable to detect complex IV (mitochondria-encoded protein MTCO1) in any of our samples. Quantification showed a decrease in complex I, II, and V expression in the SMA samples compared with controls (Figure 5, D–G).

Mitochondria are also associated with the metabolism of cholesterol, and accumulation of cholesterol in the mitochondria of the liver has been shown to impair OXPHOS (57). Metabolic dysfunction and abnormal fatty acid signaling have been previously described in SMA (58, 59), including the accumulation of lipids and cholesterol in some SMA mouse models (59). In line with these observations, in our cohort, we observed that the low-density lipoprotein (LDL) receptor–encoding gene, *LDLR*, was upregulated in SMA samples (Supplemental Figure 3F). This receptor binds to LDLs, which are the primary carriers of cholesterol in the blood. SMA patients, across all subtypes, are known to have signs of dyslipidemia, including increased blood LDL levels, and total cholesterol levels in the plasma and other tissues have been observed in some severe mouse models of SMA (59). More recently, abnormal cholesterol accumulation was found in the dystrophic muscles of DMD patients and mouse models (60), and more studies are uncovering the role of skeletal muscle and fiber type as a global modulator of cholesterol and other lipids (61). Therefore, we decided to assess the total and free cholesterol using the Cholesterol Ester-Glo bioluminescence assay system from the lysates derived from the same muscle samples. However, no differences were observed between total, free, or esterified cholesterol (Supplemental Figure 3G).

We next sought to test the number of mitochondrial DNA (mtDNA) copies in the SMA muscle samples. mtDNA copy number is used as a surrogate measure for the number of mitochondria, when such measurements are not possible. Using a previously established protocol for qPCR quantification of mtDNA copy number (62), we measured mtDNA copy number from the extracted DNA of the same samples used for sequencing. We observed that, globally, mtDNA copy number was significantly lower in SMA muscle than in control samples (Figure 5H). Furthermore, the amount of full-length *SMN* transcript was correlated to the mito-score (Supplemental Figure 3H). To test whether this was a phenomenon that was more broadly true in SMA pathophysiology, we performed the same measure on DNA extracted from the circulating PBMCs of type III patients, all of whom had been treated with nusinersen and risdiplam, and likewise noted a significant decrease in mtDNA copy number (Figure 5I). Collectively, these results suggest that mitochondrial loss is a property of SMA tissues.

This led us to question whether mitochondrial regulation was a byproduct of the loss of SMN. To test this, we utilized immortalized myoblasts derived from SMA patients (type I and II) or healthy controls (Supplemental Figure 3I). However, we did not see a difference in mtDNA copy number, despite a difference in *SMN* expression (Supplemental Figure 3J). This is true both among the SMA myoblasts, but also among different control cell lines that had different expression levels of SMN1/2 (Supplemental Figure 3I). To test whether this discrepancy with the in vivo muscle samples was due to the immaturity of the myoblasts, we performed a serum-free differentiation for 7 days to generate myotubes. It was been previously described that myoblasts increase their mtDNA copy number and number of mitochondria as part of the differentiation processes (63), and we could recapitulate this finding in our control myoblasts (Supplemental Figure 3J). The SMA myoblasts were also able to increase their mtDNA copy number to the same degree as the controls (Supplemental Figure 3J). The results mirror what was previously seen in myoblasts differentiated from SMA ESC lines, where copy number did not change, although ATP production capacity was altered (64). Taken together, these data suggest that mtDNA copy number changes are not an intrinsic feature of SMN loss, but rather a downstream consequence of muscle damage, due to either the denervation or changes that occurred to muscle maturation.

## Discussion

Here, we present a molecular characterization of a cohort of 8 SMA type II muscle tissue samples, collected from the surgical discard of adolescent patients after treatment with SMN-targeted therapies. We observed that SMN protein and RNA levels in these muscles were similar to control samples. Despite this, we detected several molecular and histological features of the muscle that are altered, including calcium regulation, fiber size, a decrease in OXPHOS complex expression, and a decrease in mtDNA copy number.

### Heterogeneity among SMA muscle samples in a small cohort.

Our study has the limitations of an observational human study, and particularly, as in many rare-disease studies, there are a limited number of patient samples. To add to the complexity of the small sample size, the type of treatment and total duration are different among patients. Unfortunately, the full natural and clinical histories of each patient were not available for all samples. This increases the complexity of interpreting the data, as not all variables can be effectively accounted for, and limits the interpretations that can be made about the effectiveness of current therapies in saving muscle function.

Furthermore, the samples are derived from surgical discards, which means the section of muscle taken may not be representative and may not be from the same spine location or depth, i.e., essentially random. Only 1 region was analyzed per patient, according to whatever was discarded during the surgery. Thus, the samples do not represent the large size of human muscles; this can also skew results both in term of fiber type and in terms of heterogeneity in atrophy or fatty infiltration in the muscle. We cannot tell how representative our fiber area calculated from a small section (Figure 2) is of the total muscle cross-sectional area, or of the total fiber number.

Despite these challenges, we were able to collect high-quality histological and transcriptional data, with minimal variation among the control samples, and to capture the expected percentage of type I versus type II fibers, suggesting that any observed heterogeneity is not due exclusively to sampling bias. Using this same approach, we were able to construct a histological and transcriptional signature of gene expression changes in type II adolescent SMA muscle. It is important to note that the SMA samples are compared to controls that are also undergoing scoliosis surgery, allowing us to find define hallmarks of SMA muscle beyond any muscle weakness changes associated with scoliosis (65). Variability was clearly higher among the SMA samples than the controls, likely reflecting the disease progression and treatment variability factors discussed above. This was particularly clear with respect to the denervation score (Figure 4). No differences were observed based on sex in this limited sample. Likely, the variability also is related to genetic modifier genes, age of onset, and time and duration of treatment, which will all need to be investigated in a study with a larger cohort size. Further sources of epigenetic variation also remain to be explored (66).

### Multiple internalized nuclei characterize a subset of SMA samples.

Another feature we observed in the SMA muscle histology was the presence of multiple centralized nuclei, present in 3 out of 5 patient samples we were able to perform histology on. The fusion, alignment, and movement of myonuclei to the periphery is a highly coordinated process, in which many players remain poorly characterized (67, 68). In 3 out of 5 patients, we observed multiple internalized, though not perfectly central, nuclei, particularly in fibers with a large diameter. To date, we are unaware of other reports of multiple internalized nuclei in SMA muscle histology, including in our own studies, although large, hypertrophic fibers with single centralized nuclei have been seen in other histological analyses (29, 69).

To determine how prevalent this phenomenon is among different types of neuromuscular diseases, we referenced the Washington University Neuromuscular atlas spinal muscular atrophy image collection (70). Indeed, muscle fibers with similar multiple internalized nuclei were present in samples from bulbospinal SMA, in a type III SMA patient (age 27), in 1 SMA low-extremely dominant (LED) patient (age 2), and a TRPV4 mutation SMA case. As the genetic origins of all these diseases are different, it suggests that the multiple internalized nuclei are not a direct consequence of *SMN1* loss, but of the resulting muscle pathology.

Based on these observations, one speculation may be that in these fibers, satellite cell fusion is an active process, perhaps in response to damage signals, but once fused the myonuclei are not able to move to the periphery because of the structural defects of cytoskeletal components, resulting in massive fiber diameters. The peripheral position of nuclei along the muscle fiber is a hallmark of skeletal muscle tissue. Actin dynamics have also been implicated in nuclear movement to the periphery, and it is known that actin assembly can organize the desmin network necessary for myofibril crosslinking (71). Differential gene expression analysis showed several cytoskeletal genes that are affected (Supplemental Table 1). However, we speculate that the mechanism responsible for these observed multinucleated fibers is not the same as in centronuclear myopathies caused by mutations in *BIN1*, *MTM1*, or *DNM2*, as classically those result in the persistence of myofibers with a single, well-centralized nucleus (72, 73).

### Involvement of SMN1 in mitochondrial homeostasis.

Mitochondria are essential organelles present in all eukaryotic cells that provide most of the ATP production via OXPHOS. This process, which occurs along the inner mitochondrial membrane, is mediated by the ETC, a series of protein complexes that turn chemical potential gradients into chemical bonds. Additionally, mitochondria serve as Ca^2+^ storage, can be a source of reactive oxygen species (ROS), sense oxygen levels, and are mediators of many cell stress and apoptosis responses. Due to the multiple roles of the mitochondria in the cell, mitochondrial dysfunction can take many different forms and can have many different causes, including problems with biogenesis, mitophagy, activity, etc. (74).

The precise role or roles of *SMN1* in mitochondrial health and homeostasis remains unclear, and it can often be difficult to tease out direct versus indirect effects (75). In the context of our study, we were only able to measure 2 aspects of mitochondrial biology, partially due to the limited amount of surgical sample and its preservation. Specifically, we measured a loss of both the RNA and protein components of the ETC, which is responsible for OXPHOS (Figure 5, A–G), as well as a decrease in the mtDNA copy number in both muscle and PBMCs from patients (Figure 5, H and I). The latter suggests, but does not confirm, a problem with either biogenesis or mitochondria or with mtDNA replication. These findings are also supported by various other reports in the literature. For example, in a muscle-specific knockout of *Smn1*, the accumulation of mitochondria with morphological abnormalities and with impaired complex I and complex IV activity and respiration deficits was observed (76). Similar results were obtained from SMA MNs (77, 78), where the authors also noted that these complex I deficiencies can cause the accumulation of ROS, which has cascading effects on other protein homeostatic processes (78). In the in vitro C2C12 model system, SMN has been proposed to target, in combination with MYOD, 2 key miRNAs for mitochondrial health: miR-1 and miR-206 (42). Similar changes in the muscle’s metabolic processes, measured by MRI after muscle stress tests, were obtained in type III and IV SMA patients (79). In particular, these MRI images showed that in the triceps, there was a disproportionate loss of the fastest contracting myofibers and decreased mitochondrial ATP synthetic function in residual white myofibers (79).

The direct or indirect role of *SMN1* in our findings is currently unclear. On the one hand, we observed a mild correlation between the total full-length SMN transcript and the mito-score in our samples (Supplemental Figure 3J). However, in myoblasts derived from type I and type II SMA patients, we do not observe a lower mitochondrial number, suggesting that the phenomenon is not directly related to *SMN1* loss, at least not in vitro (Supplemental Figure 3I). It is also possible that these changes are due to changes in the CNS or other peripheral organs, or a simple downstream consequence of the muscle atrophy associated with denervation, although in our samples, denervation and mito-score are not directly correlated (Supplemental Figure 3D). Additional factors, be they genetic or epigenetic, maybe determine the degree of mitochondrial involvement.

### Conclusion.

Taken together, the findings of this study support the need for combination therapies that target the altered pathways that we observed using our transcriptomic characterization. Although early treatment is clearly necessary for optimal treatment outcomes, not all patients are able to have these presymptomatic interventions and our data argue for the utility of combination therapies that target the *SMN*-irreversible processes (80).

## Methods

### Sex as a biological variable.

In this study, biological sex was taken into consideration. Our study sought to examine a sex-balanced cohort. In the SMA cohort, we had a sex-balanced group, with 4 female and 4 male samples. However, due limited sample availability with the controls, we had 5 female and 2 male control samples. We explicitly tested for differences in the SMA presentation based on sex, but no correlation was found between SMA subgroups for sex, and no sex effect was found among the differentially expressed genes.

### DNA extraction from human muscle samples.

DNA was extracted from approximately 2–3 mg of frozen muscle tissue using the Qiagen DNeasy Blood and Tissue Kit. Samples were first ground into a powered using a tissue grinder and then digested for 3–6 hours with proteinase K, as per the manufacturer’s recommendations.

### Relative SMN1 and SMN2 copy number calculation.

The copy number of *SMN1* and *SMN2* was determined from the previously extracted gDNA using a previously established set of primers (81). Briefly, the primers were designed to distinguish between *SMN1* and *SMN2* in exon 7 at position 6 and intron 7 at position +214. *SMN1*: forward 5′-TTTATTTTCCTTACAGGGTTT*C*-3′ and reverse 5′-GTGAAAGTATGTTTCTTCCACGTA-3′. *SMN2*: forward 5′-TTTATTTTCCTTACAGGGTTTTA-3′ and reverse 5′-GTGAAAGTATGTTTCTTCCACGCA-3′. For the relative normalizing gene, *RPPH1* (forward 5′-CGCGCGAGGTCAGACT-3′ and reverse 5′-GGTACCTCACCTCAGCCATT-3′) was used, also previously established (82). DNA was diluted to 10 ng/μL in water, and 2 μL was loaded into a 25 μL SYBR Green reaction with 0.05 μM primer. Cycling conditions were modified from the original to 50°C for 2 minutes, 95°C for 10 minutes, 45 cycles of 95°C for 15 seconds, and 58°C for 1 minute. Samples were normalized using a gDNA housekeeping gene and relative copy number was determined using the ΔΔCt method.

### mtDNA copy number calculation.

mtDNA copy number was calculated from the extracted gDNA using a previously validated protocol and primers (62). Briefly, DNA was diluted to 10 ng/μL in water, and 2 μL was loaded into a 25 μL SYBR Green reaction, as per the protocol. Samples were normalized using a gDNA housekeeping gene and relative copy number was determined using the ΔΔCt method.

### RNA extraction from human muscle samples.

RNA was extracted using the Qiagen RNeasy Mini Kit, with some modifications. Frozen samples (~3–4 mg) were ground in a tissue grinder, making sure to keep the tissue frozen. The samples were then incubated with 800 μL of RLT buffer and passed through a 20-G needle several times until the homogenized lysate was no longer viscous. Samples were then processed according to the manufacturer’s specifications. RNA quality was assessed using an Agilent TapeStation. All samples had an RIN of greater than 6. For cells, 500,000 cells were collected by spinning and resuspended in RLT buffer according to the manufacturer’s specifications.

### Real-time quantitative PCR for SMN1/2 expression.

From the RNA extracted from different cell lines, 1 μg was converted into cDNA using the High-Capacity cDNA Reverse Transcription Kit (Applied Biosystems) according to the manufacturer’s recommendation. PCR reactions (10 μL) were set up in duplicate using TaqMan Mix (Applied Biosystems) with SMN1/2 probe Hs00165806_m1 and *HPRT* as a housekeeping control gene. Relative expression was calculated using the ΔΔCt method.

### cDNA library generation and sequencing.

Libraries for sequencing were prepared using the SMART-Seq v4 PLUS Kit (Takara/CloneTech) according to the manufacturer’s specifications, using 10 ng of input RNA. Samples were sequenced after barcoding using the Illumina NovaX platform with 150-bp paired-end reads.

### Library alignment and differential gene expression analysis.

Fastq files were analyzed using the nf-core RNA-seq pipeline with the standard parameters (83). Reads were aligned to hg38. Differential expression analysis was performed using DESeq2 (84), and R was used for visualization of the data. Pathway analysis was performed using EnrichR (85) and Metascape (86).

### Fiber type deconvolution from RNA-seq data.

To determine the predicted percentage of each fiber type in our RNA-seq data, we utilized the previously published profiles from Oskolkov et al. (87).

### Derivation of mito-score and denervation scores.

Each scoring system was derived by taking the transcripts per million (TPM) quantity of the genes specified (Figure 4A or Figure 5A) and adding them together for each patient. Then, each addition was *z* scored, using the formula *z* = additive value – mean additive value of all samples divided by the standard deviation of all samples. *Z* scores were calculated using both control and SMA samples. Raw TPM values for all genes can be found in the NCBI Gene Expression Omnibus (GEO GSE252128).

### H&E staining.

Sections (12 μm) were obtained from each sample using a cryostat (Leica) and fixed in 4% paraformaldehyde for 10 minutes, followed by immersion in PBS for 2 minutes. Tissues sections were incubated in Mayer hematoxylin (Biognost, HEMH-OT-500) staining solution for 3 minutes and then in running tap water for 5 minutes followed by 30 seconds of incubation in Eosin Y (ScyTek, EYQ500). Slides were rinsed in running tap water and then dehydrated in alcohol and xylene, and then mounted using VectaMount Mounting Medium (Vector Laboratories). Images were obtained using a digital microscope (Keyence, VHX-7000).

### Sirius red staining.

Slides were washed 5 minutes with tap water and then incubated 5 minutes in 100% ethanol before letting them dry for 20 minutes. Slides were incubated in Sirius red (Sigma-Aldrich, 365548; 0.3 g in 100 mL picric acid) for 1 hour and then in running tap water (5 minutes) and acetic acid (0.5%, 5 minutes) followed by dehydration and mounting with VectaMount Mounting Medium (Vector Laboratories). Images were obtained using a digital microscope (Keyence, VHX-7000) and quantified using ImageJ software (88). All histological analyses were performed in a blinded manner.

### Cytochrome oxidase staining.

Slides were stained according to a previously established protocol, with some minor modifications (54). Briefly, slides were placed in the incubation solution consisting of 750 mg sucrose in 7.5 mL water, 2.5 mL of 0.2 M phosphate buffer, 5 mg DAB (Sigma-Aldrich, D-5637), 10 mg cytochrome *c* (Sigma-Aldrich, C-2506), and 20 μg catalase (Sigma-Aldrich, C-2506). Slides were incubated for approximately 4 hours at room temperature until some color change was evident. All slides for controls and SMA were incubated at the same time. Slides were then washed in water 3 times followed by dehydration and mounting with VectaMount Mounting Medium. Images were obtained using a digital microscope (Keyence, VHX-7000) and quantified using ImageJ software (88). All histological analyses were performed in a blinded manner

### Fiber size measurement.

ImageJ (88) was used to quantify the area in arbitrary units. Images with intact myofibers were selected, all acquired at the same Z100 ×200 objective zoom on the Keyence VHX-7000. Individual whole fibers were traced by hand and quantified using the “measure area” function. All histological analyses were performed in a blinded manner. Histograms of the area were generated using Prism 10 (GraphPad Software).

### Total cholesterol and cholesterol ester measurements from muscle samples.

Total cholesterol and cholesterol esters were measured using the Cholesterol Ester-Glo Assay (Promega, J3190) using the manufacturer’s instructions. Tissue lysate (1:25) was obtained using approximately 15 mg of tissue disrupted in 500 μL of lysis solution with a homogenizer.

### Protein extraction and Western blotting.

Protein extracts were prepared from liquid nitrogen–frozen human muscle tissues. Tissues were lysed in RIPA buffer (Sigma-Aldrich) supplemented with a protease inhibitor cocktail (Complete Mini, Roche Diagnostics). Proteins were quantified using the DC Protein Assay (Bio-Rad) and 40 μg of protein was loaded. In all blots except those using the mitochondrial complex cocktail enzyme, proteins were heated to 100°C for 5 minutes; proteins used for mitochondrial OXPHOS cocktail blots were heated to 37°C. Proteins were run in 4%–10% Bis-Tris gels (Bio-Rad) and transferred to membranes using the Turbo Transfer System (Bio-Rad). The membranes were then incubated with the corresponding antibody: anti-SMN (1:1,000; BD Biosciences, 610647), anti-vinculin (1:1,000; Sigma-Aldrich, V9191), or anti-mito cocktail (1:500; Abcam, ab110413) diluted in Tris-buffered saline containing 0.2% Tween 20 (TBST, Bio-Rad) supplemented with 5% nonfat dry milk. Primary antibody incubation was performed overnight. After 3 washes in TBST, the membranes were incubated with a horseradish peroxidase–conjugated secondary antibody (1:10,000; GE Healthcare) diluted in the blocking buffer. The membranes were further developed using the chemiluminescence substrate SuperSignal Ultra reagent (Thermo Fisher Scientific) or the SuperSignal West Femto Maximum Sensitivity Substrate (Thermo Fisher Scientific) in the case of SMN and imaged on a Bio-Rad ChemiDoc MP imaging system.

### miRNA quantification assay by real-time PCR.

miRNA quantification was performed from total RNA extractions using the TaqMan MicroRNA Reverse Transcription Kit (Applied Biosystems) according to the manufacturer’s instructions. Briefly, samples were retrotranscribed using the specific reverse transcription probe for each miRNA (Applied Biosystems: miR-1, 2222; mir-206, 000510; miR-24, 402; U6, 1973). Afterwards, each sample was quantified using a specific primer-probe set. Quantification was performed according to the ΔΔCt method, using *U6* as the housekeeping gene.

### Myoblast isolation, culture, and differentiation.

Immortalized myoblasts from control (AB1190, male, 16 years old and KM1421, female, 13 years old) and SMA (KM432 SMA I bis11PV and KM1150SMAII7PV) paravertebral muscle were obtained from the MyoLine Platform (Myology Institute, Paris, France). Cells were cultured at 37°C and 5% CO_2_ under humidity in myoblast proliferation medium (1 volume Medium 199 [Thermo Fisher Scientific, 41150020] + 4 volumes DMEM [Thermo Fisher Scientific, 61965-026] + 20% FBS [Gibco] + 50 μg/mL gentamicin [Thermo Fisher Scientific, 15750-045]) supplemented with fetuin (25 μg/mL; Life Technologies, 10344026), hEGF (5 ng/mL; Life Technologies, PHG0311), bFGF (0.5 ng/mL; Life Technologies, PHG0026), insulin (5 μg/mL, Sigma-Aldrich, 91077C-1G), and dexamethasone (0.2 μg/mL, Sigma-Aldrich, D4902). Cells were passaged with 0.05% trypsin (Thermo Fisher Scientific) and routinely tested for mycoplasma (Lonza). For any assays performed in the myoblast stage, cells were taken in the proliferation phase (~70%–80% confluent). For differentiation assays (myotubes), 24-well plates were coated with Matrigel (Corning) diluted at 1:20 in media; 150,000 cells were plated and allowed to attach for 2–5 hours, resulting in a confluent plate. The plates were then rinsed with PBS twice, and cells were changed into differentiation media (DMEM + 10 μg/mL insulin + 50 μg/mL gentamicin). Media were changed at 50% volume every 2 days during the 7-day differentiation protocol.

### Statistics.

All statistical analysis and plots for RNA, protein, and image quantifications were performed with Prism 10 using the test specified in the legend. For RNA-seq, statistical testing was done using DESeq2 with standard parameters (fold change = 1.4, *P*_adj_ ≤ 0.05, and log[fold change] of the SEM < 1.0). Reported genes are those that passed the *P*_adj_-value cutoff for multiple hypothesis testing. A *P* value of 0.05 was considered significant, and for tests with multiple hypothesis testing, a *P*_adj_ value of 0.05 or less was considered significant.

### Study approval.

Samples were obtained with patient or parental written consent, collected from the surgical residue from patients undergoing surgery for spinal sclerosis. Samples were collected under the authorization of the Minister of Research, approval number AC-2019-3502, in accordance with French and European laws.

### Data availability.

Raw RNA-seq fastq files are available in the NCBI GEO under accession number GSE252128. Differentially expressed genes are provided in Supplemental Table 1. All raw values are provided in the Supporting Data Values file.

## Author contributions

FCG designed the research study, performed experiments, analyzed and interpreted data, generated figures, and wrote the manuscript. SA, SP, EG, and SM performed experiments. MC and SV collected and preserved the muscle samples. KM derived the immortalized cells used in this study. PS designed the research study, oversaw data acquisition and interpretation, and wrote the manuscript.

## Supplementary Material

Supplemental data

Unedited blot and gel images

Supplemental tables 1-4

Supporting data values

## Figures and Tables

**Table 1:**
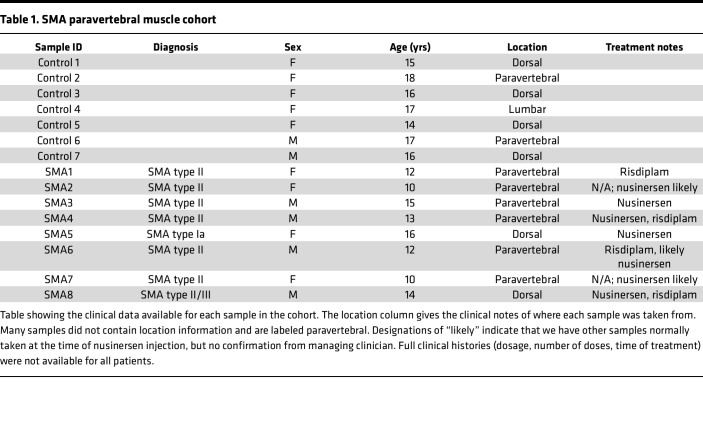
SMA paravertebral muscle cohort

**Table 2:**
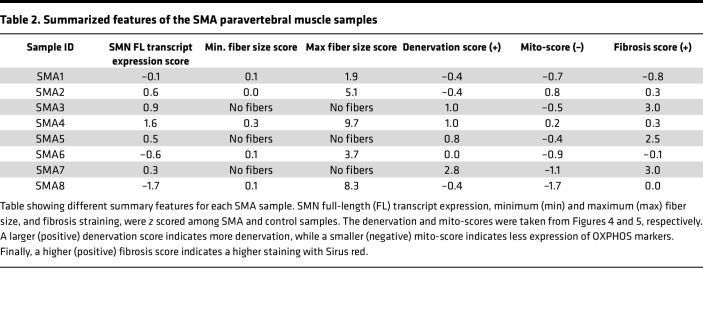
Summarized features of the SMA paravertebral muscle samples

## Version 1 09/12/2024

In-Press Preview

## Version 2 10/22/2024

Electronic publication

## References

[1] Wirth B (2020). Twenty-five years of spinal muscular atrophy research: from phenotype to genotype to therapy, and what comes next. Annu Rev Genomics Hum Genet.

[2] Mercuri E (2022). Diagnosis and management of spinal muscular atrophy: part 1: recommendations for diagnosis, rehabilitation, orthopedic and nutritional care. Neuromuscul Disord.

[3] Singh RN (2017). Diverse role of survival motor neuron protein. Biochim Biophys Acta Gene Regul Mech.

[4] Chong LC (2021). Drug discovery of Spinal Muscular Atrophy (SMA) from the computational perspective: a comprehensive review. Int J Mol Sci.

[5] Prior TW (2009). A positive modifier of spinal muscular atrophy in the SMN2 gene. Am J Hum Genet.

[6] Mailman MD (2002). Molecular analysis of spinal muscular atrophy and modification of the phenotype by SMN2. Genet Med.

[7] Groen EJN (2018). Temporal and tissue-specific variability of SMN protein levels in mouse models of spinal muscular atrophy. Hum Mol Genet.

[8] Battaglia G (1997). Expression of the SMN gene, the spinal muscular atrophy determining gene, in the mammalian central nervous system. Hum Mol Genet.

[9] Li Y-J (2020). Metabolic and nutritional issues associated with spinal muscular atrophy. Nutrients.

[10] Lipnick SL (2019). Systemic nature of spinal muscular atrophy revealed by studying insurance claims. PLoS One.

[11] Jha NN (2023). Muscle: an independent contributor to the neuromuscular spinal muscular atrophy disease phenotype. JCI Insight.

[12] Kim J-K (2020). Muscle-specific SMN reduction reveals motor neuron-independent disease in spinal muscular atrophy models. J Clin Invest.

[13] Hoy SM (2019). Onasemnogene abeparvovec: first global approval. Drugs.

[14] Chaytow H (2021). Spinal muscular atrophy: from approved therapies to future therapeutic targets for personalized medicine. Cell Rep Med.

[15] Dangouloff T, Servais L (2019). Clinical evidence supporting early treatment of patients with spinal muscular atrophy: current perspectives. Ther Clin Risk Manag.

[16] Kong L (2021). Impaired prenatal motor axon development necessitates early therapeutic intervention in severe SMA. Sci Transl Med.

[17] Ramos DM (2019). Age-dependent SMN expression in disease-relevant tissue and implications for SMA treatment. J Clin Invest.

[18] Arnold WD (2015). Spinal muscular atrophy: diagnosis and management in a new therapeutic era. Muscle Nerve.

[19] Gidaro T, Servais L (2019). Nusinersen treatment of spinal muscular atrophy: current knowledge and existing gaps. Dev Med Child Neurol.

[20] Farrar MA, Kiernan MC (2015). The genetics of spinal muscular atrophy: progress and challenges. Neurotherapeutics.

[21] Butchbach MER (2016). Copy number variations in the survival motor neuron genes: implications for spinal muscular atrophy and other neurodegenerative diseases. Front Mol Biosci.

[22] Taylor SC (2017). Droplet digital PCR versus qPCR for gene expression analysis with low abundant targets: from variable nonsense to publication quality data. Sci Rep.

[23] Chen X (2023). Comprehensive SMN1 and SMN2 profiling for spinal muscular atrophy analysis using long-read PacBio HiFi sequencing. Am J Hum Genet.

[24] Ogino S (2004). New insights on the evolution of the SMN1 and SMN2 region: simulation and meta-analysis for allele and haplotype frequency calculations. Eur J Hum Genet.

[25] Nakamori M (2017). Aberrant myokine signaling in congenital myotonic dystrophy. Cell Rep.

[26] Thomas JD (2017). Disrupted prenatal RNA processing and myogenesis in congenital myotonic dystrophy. Genes Dev.

[27] Kashima T (2007). hnRNP A1 functions with specificity in repression of SMN2 exon 7 splicing. Hum Mol Genet.

[28] Cartegni L (2006). Determinants of exon 7 splicing in the spinal muscular atrophy genes, SMN1 and SMN2. Am J Hum Genet.

[29] Millino C (2009). Different atrophy-hypertrophy transcription pathways in muscles affected by severe and mild spinal muscular atrophy. BMC Med.

[30] Folker ES, Baylies MK (2013). Nuclear positioning in muscle development and disease. Front Physiol.

[31] Kousi M, Katsanis N (2015). Genetic modifiers and oligogenic inheritance. Cold Spring Harb Perspect Med.

[33] Lamar K-M, McNally EM (2014). Genetic modifiers for neuromuscular diseases. J Neuromuscul Dis.

[34] Wirth B (2013). How genetic modifiers influence the phenotype of spinal muscular atrophy and suggest future therapeutic approaches. Curr Opin Genet Dev.

[35] Riessland M (2017). Neurocalcin delta suppression protects against spinal muscular atrophy in humans and across species by restoring impaired endocytosis. Am J Hum Genet.

[36] Oprea GE (2008). Plastin 3 is a protective modifier of autosomal recessive spinal muscular atrophy. Science.

[37] Giagnorio E (2021). MyomiRs and their multifaceted regulatory roles in muscle homeostasis and amyotrophic lateral sclerosis. J Cell Sci.

[38] Siracusa J (2018). Circulating myomiRs: a new class of biomarkers to monitor skeletal muscle in physiology and medicine. J Cachexia Sarcopenia Muscle.

[39] Horak M (2016). Muscle-specific microRNAs in skeletal muscle development. Dev Biol.

[40] Bjorkman KK (2020). miR-206 enforces a slow muscle phenotype. J Cell Sci.

[41] Przanowska RK (2020). miR-206 family is important for mitochondrial and muscle function, but not essential for myogenesis in vitro. FASEB J.

[42] Ikenaka A (2023). SMN promotes mitochondrial metabolic maturation during myogenesis by regulating the MYOD-miRNA axis. Life Sci Alliance.

[43] Chen T-H (2023). MiR34 contributes to spinal muscular atrophy and AAV9-mediated delivery of MiR34a ameliorates the motor deficits in SMA mice. Mol Ther Nucleic Acids.

[44] Abiusi E (2021). SMA-miRs (miR-181a-5p, -324-5p, and -451a) are overexpressed in spinal muscular atrophy skeletal muscle and serum samples. Elife.

[45] D’Silva AM (2023). Identification of novel CSF-derived miRNAs in treated paediatric onset spinal muscular atrophy: an exploratory study. Pharmaceutics.

[46] Magen I (2022). Muscle microRNAs in the cerebrospinal fluid predict clinical response to nusinersen therapy in type II and type III spinal muscular atrophy patients. Eur J Neurol.

[47] Giorgia Q (2023). Role of circulating biomarkers in spinal muscular atrophy: insights from a new treatment era. Front Neurol.

[48] Lin H (2022). Decoding the transcriptome of denervated muscle at single-nucleus resolution. J Cachexia Sarcopenia Muscle.

[49] Biral D (1989). Myosin heavy chain composition of muscle fibers in spinal muscular atrophy. Muscle Nerve.

[50] Lee YI (2011). Muscles in a mouse model of spinal muscular atrophy show profound defects in neuromuscular development even in the absence of failure in neuromuscular transmission or loss of motor neurons. Dev Biol.

[51] Woschitz V (2022). Mouse models of SMA show divergent patterns of neuronal vulnerability and resilience. Skelet Muscle.

[52] Sirca A, Kostevc V (1985). The fibre type composition of thoracic and lumbar paravertebral muscles in man. J Anat.

[53] Rubenstein AB (2020). Single-cell transcriptional profiles in human skeletal muscle. Sci Rep.

[54] https://neuromuscular.wustl.edu/pathol/histol/cox.htm.

[55] Rebolledo DL (2019). Denervation-induced skeletal muscle fibrosis is mediated by CTGF/CCN2 independently of TGF-β. Matrix Biol.

[56] Rath S (2021). MitoCarta3.0: an updated mitochondrial proteome now with sub-organelle localization and pathway annotations. Nucleic Acids Res.

[57] Solsona-Vilarrasa E (2019). Cholesterol enrichment in liver mitochondria impairs oxidative phosphorylation and disrupts the assembly of respiratory supercomplexes. Redox Biol.

[58] Feng Y (2023). The alterations of gut microbiome and lipid metabolism in patients with spinal muscular atrophy. Neurol Ther.

[59] Deguise M (2019). Abnormal fatty acid metabolism is a core component of spinal muscular atrophy. Ann Clin Transl Neurol.

[60] Amor F (2021). Cholesterol metabolism is a potential therapeutic target in Duchenne muscular dystrophy. J Cachexia Sarcopenia Muscle.

[61] Nomikos T (2022). The emerging role of skeletal muscle as a modulator of lipid profile the role of exercise and nutrition. Lipids Health Dis.

[62] Rooney J (2015). PCR based determination of mitochondrial DNA copy number in multiple species. Methods Mol Biol.

[63] Wagatsuma A, Sakuma K (2013). Mitochondria as a potential regulator of myogenesis. ScientificWorldJournal.

[64] Hellbach N (2018). Impaired myogenic development, differentiation and function in hESC-derived SMA myoblasts and myotubes. PLoS One.

[65] Becker L (2023). Adolescent idiopathic scoliosis is associated with muscle area asymmetries in the lumbar spine. Eur Spine J.

[66] Smeriglio P (2020). The identification of novel biomarkers is required to improve adult SMA patient stratification, diagnosis and treatment. J Pers Med.

[67] Cadot B (2015). Moving and positioning the nucleus in skeletal muscle - one step at a time. Nucleus.

[68] Roman W, Gomes ER (2018). Nuclear positioning in skeletal muscle. Semin Cell Dev Biol.

[69] https://neuromuscular.wustl.edu/pathol/sma.htm#.

[70] https://neuromuscular.wustl.edu/mother/activity.html.

[71] Roman W (2017). Myofibril contraction and crosslinking drive nuclear movement to the periphery of skeletal muscle. Nat Cell Biol.

[72] Falcone S (2014). N-WASP is required for Amphiphysin-2/BIN1-dependent nuclear positioning and triad organization in skeletal muscle and is involved in the pathophysiology of centronuclear myopathy. EMBO Mol Med.

[73] Nicot A-S (2007). Mutations in amphiphysin 2 (BIN1) disrupt interaction with dynamin 2 and cause autosomal recessive centronuclear myopathy. Nat Genet.

[74] Monzel AS (2023). Multifaceted mitochondria: moving mitochondrial science beyond function and dysfunction. Nat Metab.

[75] James R (2021). Revisiting the role of mitochondria in spinal muscular atrophy. Cell Mol Life Sci.

[76] Chemello F (2023). Dysfunctional mitochondria accumulate in a skeletal muscle knockout model of Smn1, the causal gene of spinal muscular atrophy. Cell Death Dis.

[77] Miller N (2016). Motor neuron mitochondrial dysfunction in spinal muscular atrophy. Hum Mol Genet.

[78] Thelen MP (2020). Mitochondrial defects in the respiratory complex I contribute to impaired translational initiation via ROS and energy homeostasis in SMA motor neurons. Acta Neuropathol Commun.

[79] Habets LE (2022). Magnetic resonance reveals mitochondrial dysfunction and muscle remodelling in spinal muscular atrophy. Brain.

[80] Hensel N (2020). The need for SMN-independent treatments of Spinal Muscular Atrophy (SMA) to complement SMN-enhancing drugs. Front Neurol.

[81] Feldkötter M (2002). Quantitative analyses of SMN1 and SMN2 based on real-time lightCycler PCR: fast and highly reliable carrier testing and prediction of severity of spinal muscular atrophy. Am J Hum Genet.

[82] Shebanits K (2019). Copy number determination of the gene for the human pancreatic polypeptide receptor NPY4R using read depth analysis and droplet digital PCR. BMC Biotechnol.

[83] Ewels PA (2020). The nf-core framework for community-curated bioinformatics pipelines. Nat Biotechnol.

[84] Love MI (2014). Moderated estimation of fold change and dispersion for RNA-seq data with DESeq2. Genome Biol.

[85] Chen EY (2013). Enrichr: interactive and collaborative HTML5 gene list enrichment analysis tool. BMC Bioinformatics.

[86] Zhou Y (2019). Metascape provides a biologist-oriented resource for the analysis of systems-level datasets. Nat Commun.

[87] Oskolkov N (2022). High-throughput muscle fiber typing from RNA sequencing data. Skelet Muscle.

[88] Schneider CA (2012). NIH Image to ImageJ: 25 years of image analysis. Nat Methods.

