# Supplementary material for: Characterization of SMA type II skeletal muscle from treated patients shows OXPHOS deficiency and denervation
Source: JCI Insight. 2024 Sep 12;9(20):e180992. doi: 10.1172/jci.insight.180992 (PMC11530132; doi:10.1172/jci.insight.180992)
Supplement: Supplemental data [file jciinsight-9-180992-s095.pdf]

Supplementary Information for:

Characterization of SMA Type II Skeletal Muscle from Treated Patients shows OXPHOS Deficiency and Denervation.

Fiorella Carla Grandi[1], Stéphanie Astord[1], Sonia Pezet[1], Elèna Gidaja[1], Sabrina Mazzucchi[1], Maud Chapart[2], Stéphane Vasseur[2], Kamel Mamchaoui[1], Piera Smeriglio[1*]

1. *Sorbonne Université, INSERM, Institut de Myologie, Centre de recherche en Myologie F-75013 Paris, France.*
2. Centre de Ressources Biologiques - Myobank-AFM de l'Institut de Myologie. Hôpital de la Pitié-Salpêtrière F- 75013 Paris, France.

*Correspondence should be addressed to Piera Smeriglio (piera.smeriglio@inserm.fr)

This document contains Supplemental Figures 1-3. A list of the differentially expressed genes (DEGs) can be found in **Supplementary Table 1**, which is not included in this document.

Supplemental Figure 1

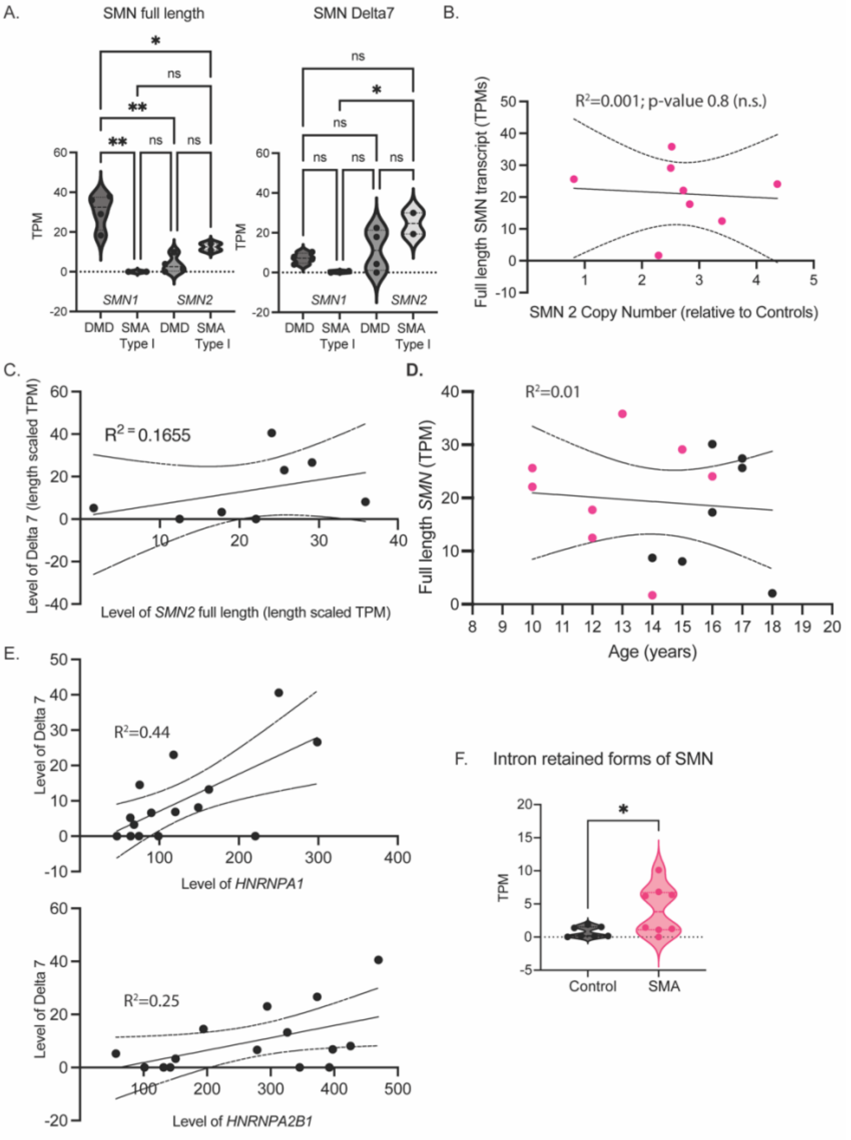

Figure S1: Regulation of *SMN* expression. (A) Reads, represented as transcripts per million (TPM) mapping to the SMN full length transcript (left) or the SMN delta 7 transcript missing exon 7 (right). The origin of full-length transcript, either the *SMN1* or *SMN2* locus, is designated below each pair of violin plots. Data was reanalyzed from GSE97806 from muscle samples of Type I SMA (n=2) or DMD (n=4). Each point represents a sample. Adjusted p-values are derived from an ordinary one-way ANOVA test with Dunnett multiple hypothesize testing; * <0.05, n.s not significant, p-value >0.05. **(B)** Correlation between the SMN copy number, dervied by qPCR from Figure 1D to the total TPMs of full-length (FL) *SMN* transript in the SMA samples (n=8). The Pearson's R^2 value and it's associated p-value are reported. The solid line represents the simple linear regression and the dashed lines represent the 95% confidence internal. (**C**) Correlation between *SMN2* transcripts per million (TPM) and Delta7 transcripts in SMA samples (n=8. The Pearson's R^2 value is reported. The solid line represents the simple linear regression and the dashed lines represent the 95% confidence internal. (**D)** Correlation between *SMN2* transcripts per million (TPM) and Delta7 transcripts in SMA samples (n=8. The Pearson's R^2 value is reported. The solid line represents the simple linear regression and the dashed lines represent the 95% confidence internal. (**D)** Correlation between the age of the patient in years and the amount of full-length *SMN* transcripts in TPM for control (n=7, black) and SMA (n=8 pink). The Pearson's R^2 value is reported. The solid line represents the simple linear regression and the dashed lines represent the 95% confidence internal. (**E**) TPMs for the delta 7 transcript in control and SMA muscle samples, compared to the TPMs of HNRNPA2A1 (top) and HNRNPA2A1 (bottom). 95% confidence intervals for the linear analysis are shown and R-squared goodness of fit values are shown. (**F**) Reads, represented as transcripts per million (TPM) mapping to the SMN transcripts with retrained introns in the n=7 paravertebral controls and n=8 SMA muscle samples. The means of each group were compared with a two-sided sudents t-test; * p-value <0.05.

Supplemental Figure 2

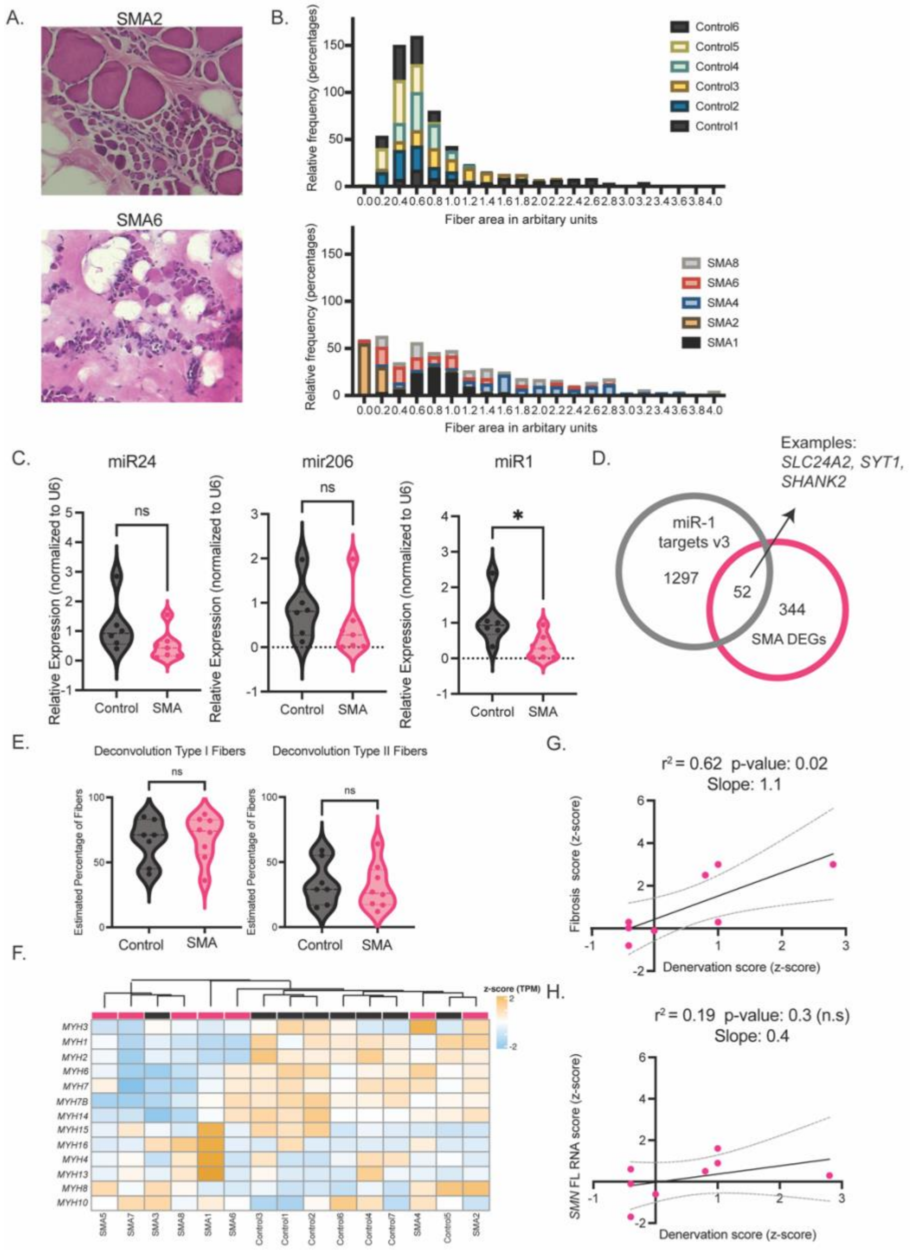

Figure S2: SMA muscle characterization through histology and transcriptomics. (A) Magnified images taken from SMA2 and SMA6 to highlight the clusters of small fibers. (**B**) Histogram of the fiber area. The frequency distribution represents the percentage of all fibers per sample that falls within the bin area. Fiber area was measured in arbitrary units. Each patient is colored in a different color. n=6 controls and n=5 SMA, with multiple fiber measurements per sample, taken from one representative image per sample. **(C)** Relative quantification (qPCR) of miR24, miR206, and miR1 respectively from total RNA. Each point represents a sample, with n=7 controls and n=8 SMA. Expression was normalized to the expression of the U6 snRNA. Comparisons between the means of the two groups was performed using a two-sided student's t-test. * <0.01. n.s. = not significant p-value >0.05. **(D)** Overlap between the predicted targets of miR-1 using miRDB with differentially expressed genes (DEGs) in SMA samples vs controls. Three example gnes related to synapse function are hilighted. **(E)** Results from the bulk-RNA sequencing fiber type deconvolution for Type I and Type II fiber signatures based on each samples' cDNA library. Each dot represents one sample's RNA-seq library (n=7 controls, n=8 SMA). The means of each group were compared with a two-sided sudents t-test; n.s. = not significant p-value >0.05. (**F**) Heatmap of the expression of myosin heavy chains in each sample. Hierarchical clustering is based only on myosin expression. Transcripts per million are presented as a z-score. (**G-H**) Correlation between the denervation score (derived from Figure 4, Table 2) and the fibrosis score (G, derived from Table 2) or the SMN full length (FL) transcript amount (H, dervied from Table 2) for the 8 SMA samples in pink. The Pearson's R^2 value and it's associated p-value are reported. The solid line represents the simple linear regression and the dashed lines represent the 95% confidence internal.

Supplemental Figure 3

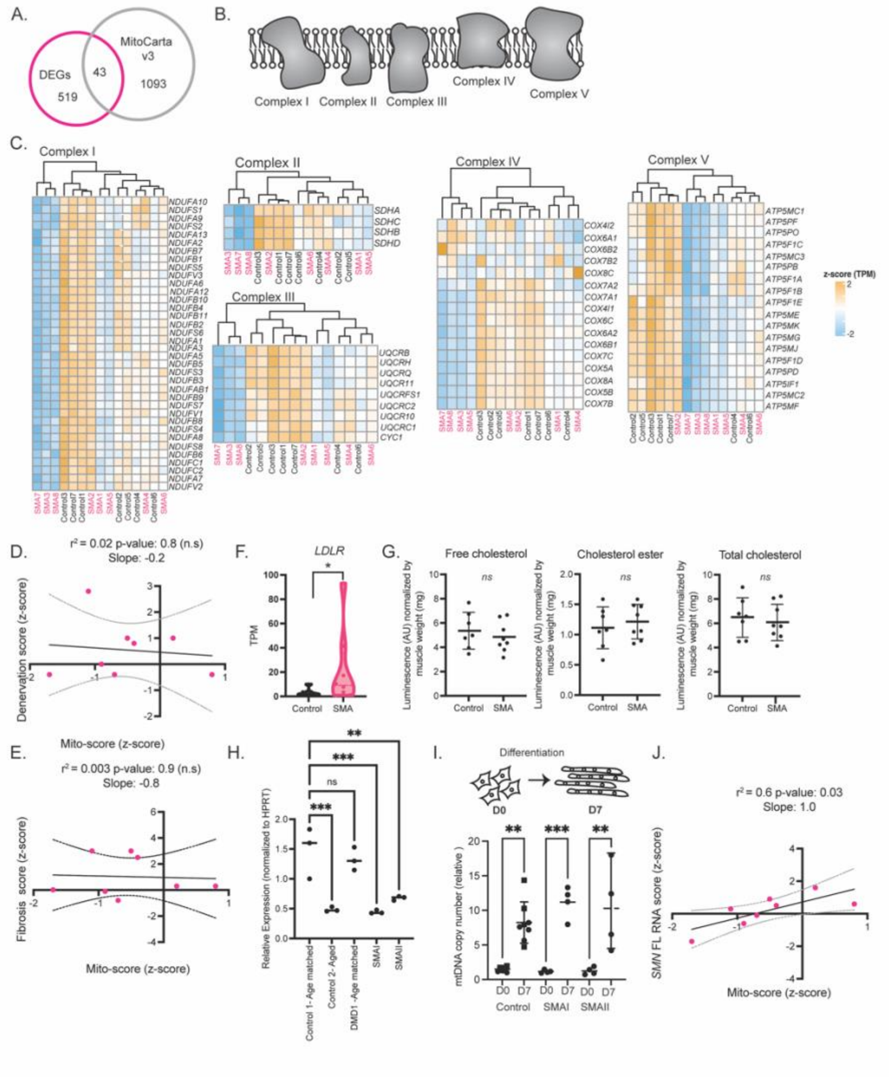

Figure S3: Changes in OXPHOS transcription and mtDNA copy number in SMA.
A. Overlap between the genes known to be localized in the mitochondria, from MitoCarta v3 compared to the differentially expressed genes in SMA Type II muscle. **B.** Scheme of each of the complexes of the ETC, with the corresponding genes diagrammed in the heatmaps in C. **(C)** Heatmaps showing the expression in TPMs for each gene that forms complexes 1-5. Hierarchical clustering was performed based on the expression of each subset of complex genes. TPMs are z-scored across the row. (**D-E**) Correlation between mito-score (derived from Figure 5, Table 2) and the denervation score (D, derived from Table 2 and Figure 4) or the fibrosis score(E, dervied from Table 2) for the 8 SMA samples in pink. The Pearson's R^2 value and it's associated p-value are reported. The solid line represents the simple linear regression and the dashed lines represent the 95% confidence interval. **(F)** Transcript per million (TPM) counts for *LDLR* from the RNA-sequencing of muscle samples. Each point represents one patient sample (n=7 controls, n=8 SMA). P-values are based on the adjusted p-values derived from DeSEQ2 which considers multiple hypothesis testing. **(G)** Results from the Cholesterol-Glo luciferase assay. Each point represents one muscle lysate. Group means were compared using a two-sided students-test test (n=7 controls and n=8 SMA); n.s. = not significant, p-value >0.05**.** (**H**) Real time quantitative PCR of *SMN1/2* transcript expression in myoblasts using a Taqman probe that detects transcripts from both loci. SMA cells were compared to both age-matched control (10-16 years old) and DMD1 sample, as well as to an older non-aged matched control (50-63 years old). (**J**) Correlation between mito-score (derived from Figure 5, Table 2) and the denervation score (D, derived from Table 2 and Figure 4) and the SMN full length (FL) transcript amount (H, dervied from Table 2) for the 8 SMA samples in pink. The Pearson's R^2 value and it's associated p-value are reported. The solid line represents the simple linear regression and the dashed lines represent the 95% confidence interval.
