# Supplementary material for: Characterization of SMA type II skeletal muscle from treated patients shows OXPHOS deficiency and denervation
Source: JCI Insight. 2024 Sep 12;9(20):e180992. doi: 10.1172/jci.insight.180992 (PMC11530132; doi:10.1172/jci.insight.180992)
Supplement: Unedited blot and gel images [file jciinsight-9-180992-s096.pdf]

Uncropped Western Blot Images

“Characterization of SMA Type II Skeletal Muscle from Treated Patients shows OXPHOS Deficiency and Denervation.”

Grandi et al. 2024 (Smerilgio)

Figure 1G

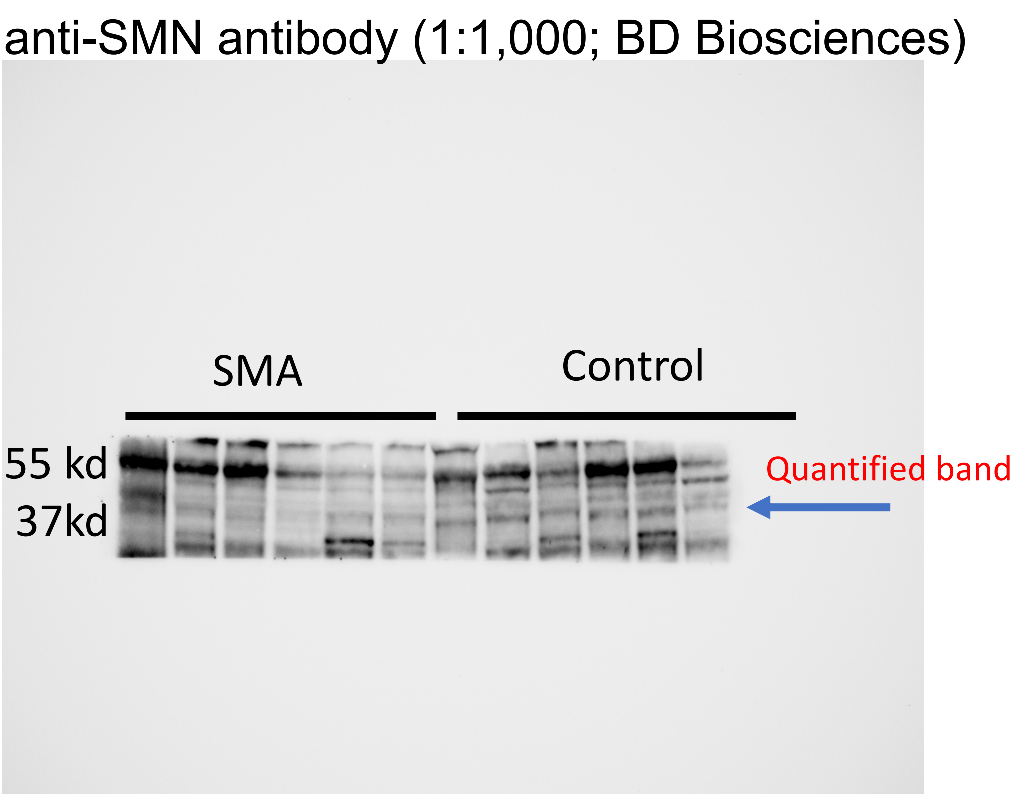

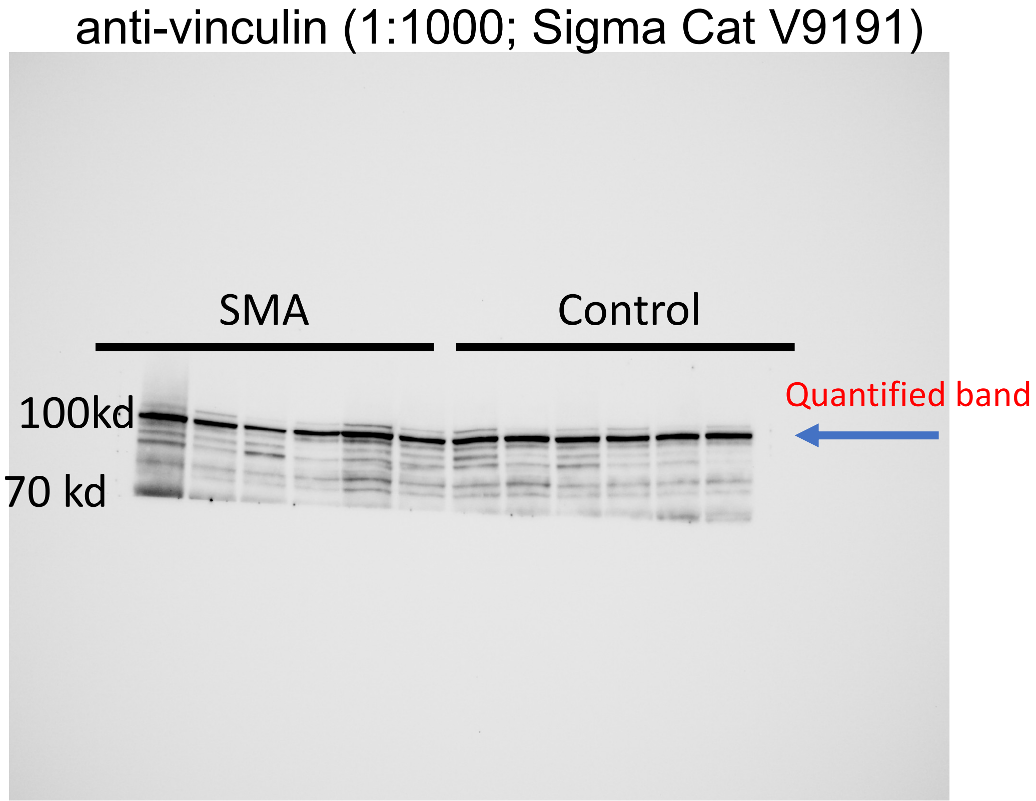

Note: WB membranes are cut after transfer and before AB incubation. Images represent uncropped images of the cut membranes.

Figure 5C

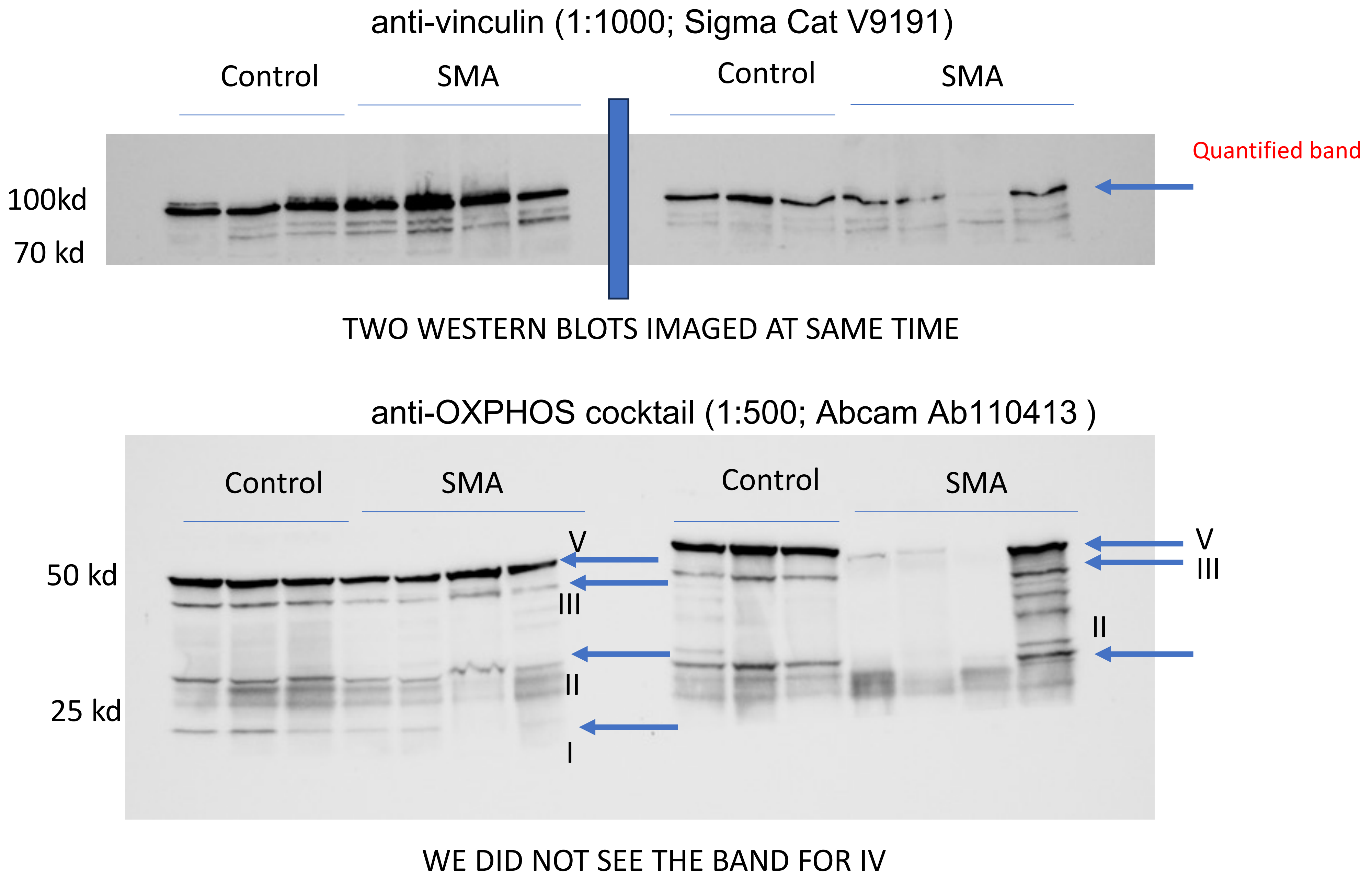
anti-vinculin (1:1000; Sigma Cat V9191)
Control
SMA
Control
SMA
100kd
70 kd
Quantified band
TWO WESTERN BLOTS IMAGED AT SAME TIME
anti-OXPHOS cocktail (1:500; Abcam Ab110413)
Control
SMA
Control
SMA
50 kd
25 kd
V
III
II
I
V
III
II
WE DID NOT SEE THE BAND FOR IV
